# Impact of 100 LRRK2 variants linked to Parkinson's disease on kinase activity and microtubule binding

**DOI:** 10.1042/BCJ20220161

**Published:** 2022-09-06

**Authors:** Alexia F. Kalogeropulou, Elena Purlyte, Francesca Tonelli, Sven M. Lange, Melanie Wightman, Alan R. Prescott, Shalini Padmanabhan, Esther Sammler, Dario R. Alessi

**Affiliations:** 1 MRC Protein Phosphorylation and Ubiquitylation Unit, School of Life Sciences, University of Dundee, Dow Street, Dundee, U.K.; 2 Aligning Science Across Parkinson's (ASAP) Collaborative Research Network, Chevy Chase, MD 20815, U.S.A.; 3 Dundee Imaging Facility, School of Life Sciences, University of Dundee, Dundee DD1 5EH, U.K.; 4 The Michael J. Fox Foundation for Parkinson's Research, New York, NY, U.S.A.; 5 Molecular and Clinical Medicine, Ninewells Hospital and Medical School, University of Dundee, Dundee DD1 9SY, U.K.

**Keywords:** G-proteins, leucine-rich repeat kinase, Parkinson's disease, signaling

## Abstract

Mutations enhancing the kinase activity of leucine-rich repeat kinase-2 (LRRK2) cause Parkinson's disease (PD) and therapies that reduce LRRK2 kinase activity are being tested in clinical trials. Numerous rare variants of unknown clinical significance have been reported, but how the vast majority impact on LRRK2 function is unknown. Here, we investigate 100 LRRK2 variants linked to PD, including previously described pathogenic mutations. We identify 23 LRRK2 variants that robustly stimulate kinase activity, including variants within the N-terminal non-catalytic regions (ARM (E334K, A419V), ANK (R767H), LRR (R1067Q, R1325Q)), as well as variants predicted to destabilize the ROC:COR_B_ interface (ROC (A1442P, V1447M), COR_A_ (R1628P) COR_B_ (S1761R, L1795F)) and COR:COR dimer interface (COR_B_ (R1728H/L)). Most activating variants decrease LRRK2 biomarker site phosphorylation (pSer935/pSer955/pSer973), consistent with the notion that the active kinase conformation blocks their phosphorylation. We conclude that the impact of variants on kinase activity is best evaluated by deploying a cellular assay of LRRK2-dependent Rab10 substrate phosphorylation, compared with a biochemical kinase assay, as only a minority of activating variants (COR_B_ (Y1699C, R1728H/L, S1761R) and kinase (G2019S, I2020T, T2031S)), enhance *in vitro* kinase activity of immunoprecipitated LRRK2. Twelve variants including several that activate LRRK2 and have been linked to PD, suppress microtubule association in the presence of a Type I kinase inhibitor (ARM (M712V), LRR (R1320S), ROC (A1442P, K1468E, S1508R), COR_A_ (A1589S), COR_B_ (Y1699C, R1728H/L) and WD40 (R2143M, S2350I, G2385R)). Our findings will stimulate work to better understand the mechanisms by which variants impact biology and provide rationale for variant carrier inclusion or exclusion in ongoing and future LRRK2 inhibitor clinical trials.

## Introduction

**Figure 1.**
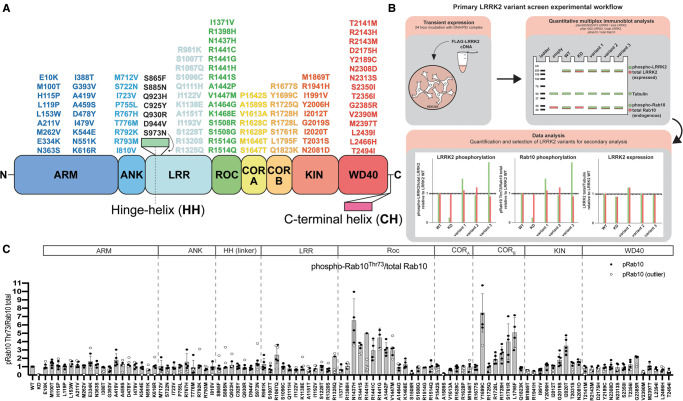
Domain location of 100 LRRK2 variants and experimental workflow to assess LRRK2 variant activity by quantitative immunoblotting. (A) LRRK2 domain structure highlighting 100 PD and CD-associated variants within the armadillo (ARM), ankyrin (ANK), leucine rich repeats (LRR), Ras of complex proteins (ROC), C-terminal of ROC A and B (COR_A_, COR_B_), kinase (KIN), and WD40 domains. The LRRK2 variants located in the linker region between the HH and LRR domain are listed in black. (B) Workflow schematic outlining the characterization of the selected LRRK2 variants in a HEK293 overexpression system, followed by quantitative immunoblotting and quantitation of LRRK2 activity relative to wildtype LRRK2. (C) FLAG-tagged LRRK2 wildtype, kinase dead (KD = D2017A), and the indicated variants were transiently expressed in HEK293 cells. Twenty-four hours post-transfection, cells were lysed and analyzed by quantitative immunoblotting (as in Supplementary Figure S2A). Quantified immunoblotting data are presented as ratios of pRab10^Thr73^/total Rab10, normalized to the average of LRRK2 wildtype values for each replicate (mean ± SD). Combined immunoblotting data from up to six independent biological replicates are shown. Dashed lines segment the graphs into corresponding regions of LRRK2 as listed in the domain schematic.

One-to four percent of all cases of Parkinson's disease (PD) are caused by genetic changes in leucine-rich repeat kinase-2 (LRRK2) [1–3]. Additionally, LRRK2 has been linked to modify risk for Crohn's disease (CD) [4]. LRRK2 is a large multidomain enzyme that forms multimeric species [5,6]. It consists of an N-terminus armadillo (ARM), ankyrin (ANK) and leucine-rich repeats (LRR), followed by a C-terminal Roco type GTPase, protein kinase and WD40 domain [7] (Figure 1A). The Roco GTPase domain consists of three subdomains, namely a ROC GTPase followed by two scaffolding domains termed COR_A_ and COR_B_. High-resolution Cryo-EM structures of full length [8], as well as the catalytic C-terminal moiety of LRRK2, have been solved in which the catalytic kinase domain is in an inactive open conformation, [9] and more recently in a closed conformation [10]. These structures have provided major insights into the overall structure and function of LRRK2.

LRRK2 is activated following its recruitment to cellular membranes via interactions with Rab29 and other Rab GTPases, likely via its N-terminal ARM domain [11–15]. LRRK2 phosphorylates a subgroup of Rab GTPases at membranes, including Rab8A and Rab10, at a conserved Ser/Thr residue located within the effector binding Switch-II domain [16–18]. This phosphorylation does not impact intrinsic Rab GTPase activity but promotes binding to a new set of effectors, including RILPL1/2 and JIP3/JIP4 [17,19–22]. Interaction of LRRK2-phosphorylated Rab8A and Rab10 with RILPL1 interferes with ciliogenesis in brain cholinergic neurons in the striatum, decreasing their ability to sense Sonic hedgehog in a neuro-protective circuit that supports dopaminergic neurons [19].

LRRK2-phosphorylated Rab proteins are dephosphorylated by the PPM1H phosphatase [23]. At least in overexpression studies, certain pathogenic mutations, as well as treatment with selective Type I kinase inhibitors that promote the LRRK2 kinase domain to adopt an active conformation, induce helical oligomerization of LRRK2 on microtubule filaments [24–27]. This has been proposed to disrupt vesicle trafficking by causing a ‘roadblock' for microtubule-based motors [6,9]. The closed, active conformation of LRRK2 also leads to the dephosphorylation of a cluster of phosphorylation sites (Ser910, Ser935, Ser955 and Ser973) located between the hinge-helix (HH) and LRR domain through an unknown mechanism [28–30]. Certain pathogenic mutations such as G2019S (located within the kinase domain) promote autophosphorylation of LRRK2 at Ser1292 [31].

Seven missense mutations located within the ROC (N1437H, R1441G/C/H), COR_B_ (Y1699C) and kinase (G2019S, I2020T) domains have been well-characterized and ascertained to stimulate LRRK2 kinase activity and cause PD [3,32]. The G2019S mutation that substitutes a glycine for serine within the magnesium-binding DYG motif is by far the most frequent PD-associated LRRK2 mutation [33,34]. In addition, a variant located within the WD40 domain (G2385R), is common in Chinese Han and Taiwanese populations and moderately increases PD risk; biochemical analysis suggests that G2385R blocks WD40 dimerization and moderately enhances LRRK2 kinase activity [35–37]. Over 1000 rare variants of LRRK2 have been reported [38–40], and a recent study employed a computational tool that predicts a Parkinson's pathogenic ‘REVEL score' for each variant, with a score >0.600 predicted to be pathogenic [41–43].

Here, we describe a robust workflow to experimentally evaluate LRRK2 variant impact on LRRK2 function. Specifically, we utilize LRRK2-dependent Rab10 phosphorylation at Thr73 as a readout for the LRRK2 kinase pathway activity (pRab10^Thr73^), using selective phospho-specific antibodies [44]. From amongst 100 LRRK2 variants, we identified 23 that robustly enhance LRRK2 kinase activity, defined as >1.5-fold above LRRK2 wildtype. These include novel variants within the N-terminal, non-catalytic ARM, ANK and LRR regions, as well as within the ROC and COR_B_ domains, that are predicted to destabilize the interface between the ROC and COR_B_ domains or impact the COR:COR dimer interface. Amongst the 100 variants tested, we also report a subset of 12 variants that suppress the ability of LRRK2 to bind microtubules in the presence of a Type I LRRK2 kinase inhibitor. Overall, our work will assist in the interpretation of the many reported LRRK2 variants of unknown clinical significance identified in individuals and families with PD [41], by informing on the variant impact on LRRK2 function and by providing a framework for the thorough functional characterization and cataloging of other LRRK2 variants of unknown significance. In fact, the functional stratification of LRRK2 variants is particularly important in view of targeted treatments such as LRRK2 kinase inhibitors entering clinical trials.

## Results

### Selection of LRRK2 variants

One hundred LRRK2 variants were selected from previous genetic analyses of PD patients (Supplementary Table S1). This list includes the seven ‘definitely pathogenic’ mutations as listed in the MDSgene database (https://www.mdsgene.org) (ROC (N1437H, R1441), COR_B_ (Y1699C), kinase (G2019S, I2020T)) as well as two previously characterized variants, including the kinase (T2031S) and WD40 (G2385R) variants that activate LRRK2 (Figure 1A). A variant linked to increased CD disease risk and LRRK2 activation (kinase (N2081D)), as well as variants reported to protect from PD and CD (ARM (N551K) and ROC (R1398H)), were also included [4]. Other than the well-characterized variants mentioned above, the remainder have only been reported in a single or small number of cases and studies and often without clear evidence of pathogenicity in line with current guidelines [45]. Literature citations and REVEL scores [41] (http://database.liulab.science/dbNSFP) for each of the selected variants, as well as evolutionary conservation scores for each variant amino acid determined using the Consurf database (https://consurf.tau.ac.il/) [46], are tabulated in Supplementary Table S1. The selected variants are located within the following domains: ARM (18), ANK (9), LRR (12), ROC (15), COR_A_ (7), COR_B_ (8), kinase (9) and WD40 (15) domains, as well as between the boundaries of the ANK and LRR (5) and LRR and ROC domains (2) (Figure 1A).

### Impact of variants on LRRK2 activity in a cellular assay

To assess the impact of each variant, we utilized a HEK293 cell overexpression system (summarized in Figure 1B) and assessed LRRK2-mediated pRab10^Thr73^, LRRK2 autophosphorylation at Ser1292, as well as LRRK2 biomarker site phosphorylation (Ser935, Ser955 and Ser973). HEK293 cells lend themselves for the interrogation of LRRK2-dependent pRab10^Thr73^ as they have low levels of endogenous LRRK2 but high endogenous Rab10 expression with resulting complete lack of phosphorylation at the LRRK2-dependent Rab10^Thr73^ phospho-site. The wildtype and all LRRK2 variant constructs used in this study contain the common S1647T variant [47]. The S1647T variant is observed in ∼30% of alleles listed in the gnomAD database (280 000 human alleles) [48] and the Parkinson's disease (PD) variant Browser (103 000 human alleles) [49], with no difference between cases and controls. Our data reveal that the T1647 compared with S1647 variant does not impact LRRK2 activity in wildtype, R1441G and G2019S backgrounds (Supplementary Figure S1).

**Figure 2.**
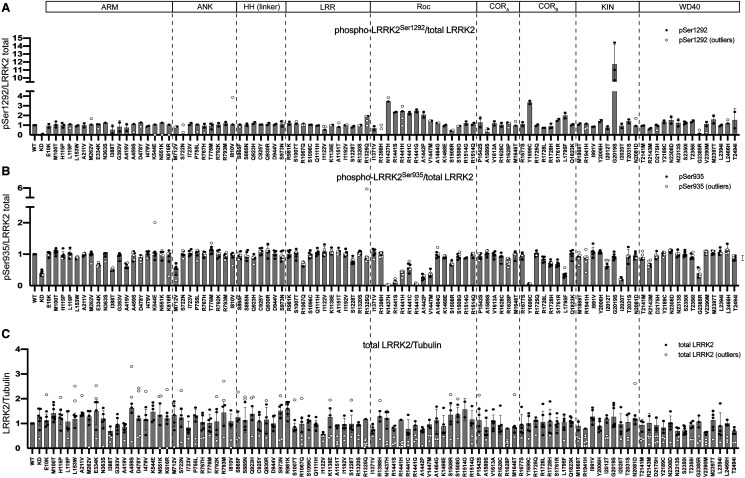
Quantitative analysis of phosphorylation and expression of selected PD and CD-associated LRRK2 variants assessed in primary screens. FLAG-tagged LRRK2 wildtype, kinase dead (KD = D2017A), and the indicated variants were transiently expressed in HEK293 cells. Twenty-four hours post-transfection, cells were lysed and analysed by quantitative immunoblotting (as in Supplementary Figure S2A). Quantified immunoblotting data are presented as ratios of phospho-LRRK2 Ser1292/total LRRK2 (**A**), phospho-LRRK2 Ser935 (**B**), and total LRRK2/Tubulin (**C**), normalized to the average of LRRK2 wildtype values for each replicate (mean ± SD). Combined immunoblotting data from six independent biological replicates are shown. Dashed lines segment the graphs into corresponding regions of LRRK2 as listed in the domain schematic.

**Figure 3.**
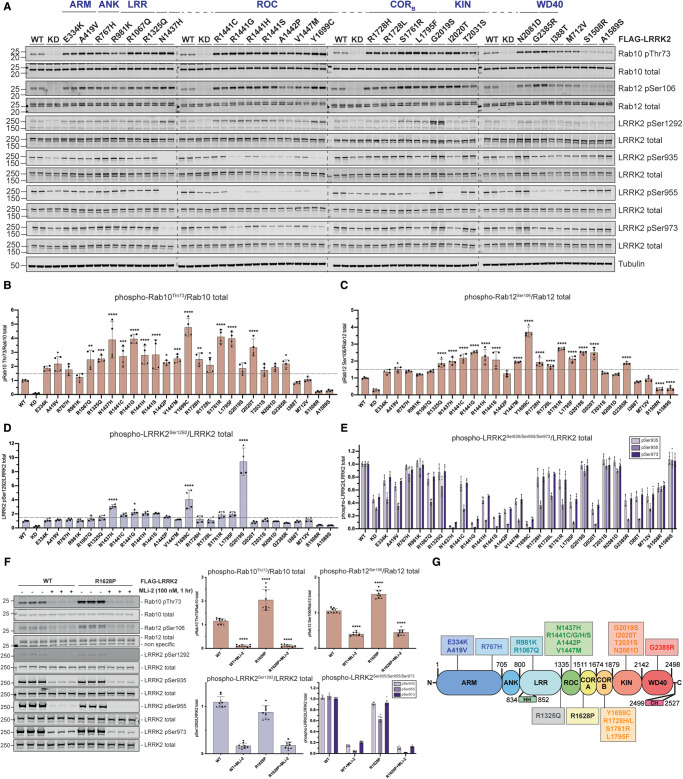
23 LRRK2 variants with mutations spanning multiple domains significantly augment LRRK2-mediated Rab10^Thr73^ phosphorylation. (**A**) FLAG-tagged LRRK2 wildtype, kinase dead (KD = D2017A) and the indicated variants were transiently expressed in HEK293 cells. Twenty-four hours post-transfection, cells were lysed and analysed by quantitative immunoblotting using the indicated antibodies. Each lane represents a different dish of cells. Data quantification is shown in (**B**–**E**). (**B**–**E**) Quantified immunoblotting data are presented as ratios of pRab10 Thr73/total Rab10 (**B**), pRab12 Ser106/total Rab12 (**C**), phospho-LRRK2 Ser1292/total LRRK2 (**D**), phospho-LRRK2 Ser935/total LRRK2, phospho-LRRK2 Ser955/total LRRK2, or phospho-LRRK2 Ser973/total LRRK2 (**E**), normalized to the average of LRRK2 wildtype values for each replicate (mean ± SD). Combined immunoblotting data from two independent biological replicates (each performed in duplicate) are shown. Data were analysed using one-way ANOVA with Dunnett's multiple comparisons test. Statistical significance was determined from four replicate values for each variant, and represented with *P*-values (*P < 0.05, **P < 0.01, ***P < 0.001, ****P < 0.0001). (**F**) FLAG LRRK2 WT or R1628P was expressed in HEK293 cells. Each lane represents a different dish of cells. One hour prior to lysis, cells were treated with vehicle (0.1% v/v DMSO) or 100 nM MLi-2. Cell lysates were analysed by quantitative immunoblotting and quantified data are analysed and presented as in (**A**–**E**). Quantified data are representative of three independent experiments, each performed in triplicate. (**G**) Domain schematic of LRRK2 highlighting the position of the 23 LRRK2 variants selected for further analysis.

In a primary screen, the selected variants were analyzed in parallel and normalized to the effect of the LRRK2 wildtype protein, and data were merged from up to six independent screens (Figures 1C and 2, Supplementary Figures S2 and S3). For each immunoblot analysis, we also included LRRK2 wildtype for normalization and a kinase-inactive LRRK2[D2017A] variant as a negative control for kinase activity. In an additional experiment, we performed a screen with 98 variants treated ±MLi-2 LRRK2 inhibitor [50] and found that this compound suppressed Rab10 phosphorylation in all cases, thereby demonstrating that activity measured is indeed mediated by LRRK2 (Supplementary Figure S4). LRRK2 variant impact on LRRK2 kinase activity was defined as ‘activating’ if pRab10^Thr73^ levels were >1.5-fold and ‘reduced’ if pRab10^Thr73^ levels were <0.5-fold relative to the LRRK2 wildtype protein. Our analysis highlighted 23 variants (ARM (E334K, A419V), ANK (R767H), LRR (R981K, R1067Q), boundary between LRR and ROC (R1325Q), ROC (**N1437H**, **R1441G/C/H/S**, A1442P, V1447M), COR_B_ (**Y1699C**, **R1728H**/L, S1761R, L1795F), kinase (**G2019S**, **I2020T**, **T2031S**, **N2081D**) and WD40 (**G2385R**)), that enhance LRRK2-mediated Rab10 phosphorylation (Figure 1C, Supplementary Figure S2). Twelve of these variants (shown in bold) had previously been reported to stimulate LRRK2 kinase activity. Using the same overexpression system, we then reanalyzed all 23 activating variants from the primary screen in a secondary quantitative immunoblot analysis, in which we confirmed 22 of the 23 variants to robustly enhance LRRK2-mediated Rab10^Thr73^ phosphorylation >1.5-fold above wildtype (Figure 3). Only the LRRK2 R981K variant fell below the 1.5-fold cut-off and could not be confirmed to be activating in the secondary screen (Figure 3). The activity of the R1628P variant which resides in the COR_A_ domain was analyzed separately from the group of other activating variants and was found to stimulate Rab10 phosphorylation ∼2-fold (Figure 3F). We also performed the secondary screen monitoring LRRK2-mediated pRab12^Ser106^ phosphorylation instead of pRab10^Thr73^ phosphorylation (Figure 3A,C). This analysis revealed that most activating variants also enhanced Rab12 phosphorylation in a LRRK2 kinase-dependent manner at Ser106. However, we observed three variants (A1442P (ROC), T2031S (kinase) and N2081D (kinase)) that enhanced Rab10 but not Rab12 phosphorylation (Figure 3A–C). Thus, from the group of 100 LRRK2 variants analyzed, we conclude that 23 activate (based on Rab10 phosphorylation), 75 have no effect and 2 variants (ROC (S1508R) and COR_A_ (A1589S)) significantly reduce LRRK2 kinase activity.

The G2019S mutation stimulated LRRK2 Ser1292 autophosphorylation ∼10-fold, to a greater extent than other variants (Figures 2A and 3A,D, Supplementary Figure S2). Nine variants (ROC (N1437H, R1441G/C/S/H, A1442P) and COR_B_ (Y1699C, S1761R, L1795F)), increased Ser1292 autophosphorylation 2- to 4-fold (Figures 2A and 3A,D, Supplementary Figure S2).

Previous work revealed that variants that stimulate LRRK2 kinase activity, such as ROC (R1441G/C) and COR_B_ (Y1699C), suppressed LRRK2 biomarker phosphorylation, likely by promoting the closed, active conformation of the LRRK2 kinase domain [28–30]. Consistent with this, 10 activating variants (ROC (N1437H, R1441G/H/S, A1442P, V1447M), COR_B_ (Y1699C, L1795F), kinase (I2020T) and WD40 (G2385R)), displayed >2-fold reduction in phosphorylation of all biomarker sites (Figures 2B and 3A,E, Supplementary Figure S3). Seven variants ((ARM (E334K and A419V), LRR (R1067Q), ROC (R1441C) and COR_B_ (R1728H/L and S1761R)) showed reduced Ser955 phosphorylation, with a moderate impact on Ser935 and Ser973 phosphorylation (Figures 2B and 3A,E, Supplementary Figures S2 and S3). The reduced activity variants (ROC (S1508R) and COR_A_ (A1589S)), possessed similar biomarker phosphorylation as wildtype LRRK2 (Figures 2B and 3A,F, Supplementary Figures S2 and S3). Two variants (ARM (I388T) and ANK (M712V)) decreased biomarker site phosphorylation without impacting LRRK2-dependent pRab10^Thr73^ phosphorylation (Figures 1C, 2B and 3A,F, Supplementary Figures S2 and S3). Although the majority of the 23 variants that stimulate LRRK2 activity reduce biomarker site phosphorylation, variants located within the ANK (R767H), LRR (R1325Q) or kinase (G2019S, T2031S and N2081D) domains do not reduce Ser935 or other biomarker sites. None of the variants studied increased the basal level of phosphorylation of the biomarker sites.

We observed that the R1398H protective variant [51] displayed similar pRab10^Thr73^, pLRRK2^Ser1292^ and LRRK2 biomarker phosphorylation levels compared with that of wildtype LRRK2 (Figures 1C and 2, Supplementary Figures S2 and S3). We also analyzed the effect of the R1398H mutation on the kinase activity of three different LRRK2 pathogenic variants (R1441G, Y1699C and G2019S) and found that this mutation does not significantly reduce LRRK2 activity within these variants (Supplementary Figure S5). Most variants analyzed did not markedly impact LRRK2 expression levels in HEK293 cells (Figure 2C, Supplementary Figure S2).

### Impact of variants in an immunoprecipitation *in vitro* assay

**Figure 4.**
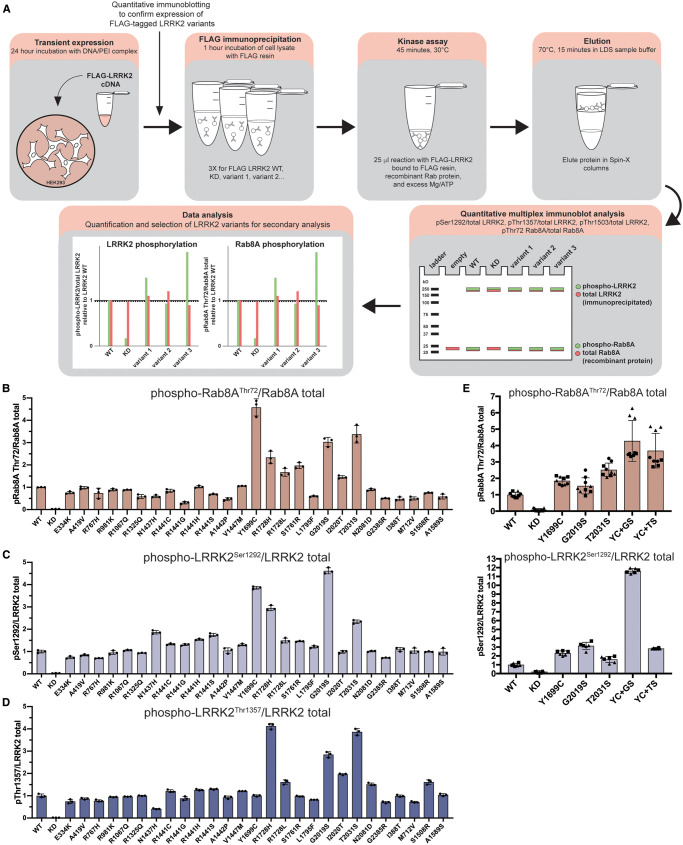
COR_B_ and kinase domain LRRK2 variants enhance *in vitro* LRRK2 kinase activity against recombinant Rab8A. (**A**) Workflow schematic outlining the immunoprecipitation kinase assay method employed to assess *in vitro* kinase activity of LRRK2 variants against recombinant Rab8A. Kinase reaction products were analysed by quantitative immunoblotting (as in Supplementary Figure S6–S8). (**B**–**D**) Data obtained from quantitative immunoblotting analysis of FLAG-LRRK2 immunoprecipitation kinase reactions for the indicates variants are presented as ratios of pRab8A^Thr72^/total Rab8A (**B**), phospho-LRRK2 Ser1292/total LRRK2 (**C**), and phospho-LRRK2 Thr1357/total LRRK2 (**D**) normalized to the average of LRRK2 wildtype values (mean ± SD). (**E**) Data obtained from quantitative immunoblotting analysis of FLAG-LRRK2 immunoprecipitation kinase reactions for the indicates variants are presented as ratios of pRab8A^Thr72^/total Rab8A, phospho-LRRK2 Ser1292/total LRRK2, relative to the average of LRRK2 wildtype values (mean ± SD).

Previous work revealed that pathogenic LRRK2 variants, such as the common G2019S kinase domain variant, directly enhance LRRK2 kinase activity, and this effect was recapitulated in recombinant *in vitro* kinase assays [52,53]. In contrast, other pathogenic variants, such as ROC (R1441G), despite enhancing LRRK2 kinase pathway activity to a greater extent than the G2019S variant *in vivo*, failed to stimulate the kinase activity of recombinant LRRK2 *in vitro* [53]*.* The contrasting effects on *in vitro* kinase activity suggest that these variants activate LRRK2 in cells by a different mechanism. This prompted us to investigate which of the *in vivo* activating variants enhanced the activity of recombinant LRRK2 in an *in vitro* kinase assay. We expressed and immunopurified FLAG-tagged wildtype or mutant LRRK2 in HEK293 cells and subjected the purified protein to an *in vitro* kinase assay employing recombinant Rab8A as a substrate (Figure 4A). LRRK2-mediated phosphorylation of Rab8A at Thr72 was quantified using a previously characterized pan-selective phospho-Rab antibody [44] (Figure 4A). We observed that of the 22 variants which were found to enhance LRRK2 kinase activity as measured by pRab10^Thr73^ in the cellular assay, only 7 enhanced Rab8A phosphorylation *in vitro* by >1.5-fold relative to wildtype LRRK2 (COR_B_ (Y1699C, R1728H, R1728L, S1761R) and kinase (G2019S, I2020T and T2031S)) (Figure 4B, Supplementary Figures S6 and S7). The kinase G2019S variant enhanced *in vitro* Rab8A phosphorylation around 3-fold, while the COR_B_ Y1699C and kinase T2031S variants stimulated activity ∼4-fold (Figure 4B, Supplementary Figures S6 and S7). None of the variants within the ARM, ANK, LRR, ROC or WD40 domains enhanced immunoprecipitated LRRK2 activity *in vitro* (Figure 4B, Supplementary Figure S6). The variants displaying reduced activity in the cellular assay (ROC (S1508R) and COR_A_ (A1589S)) possessed similar *in vitro* kinase activity towards Rab8A as the immunoprecipitated wildtype LRRK2 (Figure 4B, Supplementary Figure S6), suggesting that these may impact LRRK2 kinase pathway activity in cells by an indirect mechanism rather than having a direct effect on LRRK2 kinase activity. Four variants (COR_B_ (Y1699C, R1728H) and kinase (G2019S, T2031S)) enhanced Ser1292 autophosphorylation over 2-fold in an *in vitro* assay (Figure 4C, Supplementary Figures S6 and S7). We also studied autophosphorylation of LRRK2 at Thr1357 [54] and Thr1503 [55] employing recently developed phospho-antibodies. This revealed that the COR_B_ (R1728H), as well as the three kinase variants (G2019S, I2020T and T2031S), enhanced autophosphorylation of these sites ∼2- to 4-fold (Figure 4D, Supplementary Figures S6 and S7). Since both COR_B_ and kinase domain variants increase LRRK2 activity in an immunoprecipitation assay, we next explored the impact of combining COR_B_ and kinase domain activating variants on LRRK2 kinase activity *in vitro*. This revealed that the Y1699C + T2031S as well as the Y1699C + G2019S combination increased LRRK2-mediated Rab8A phosphorylation to a greater degree than individual mutations assayed in parallel experiments (Figure 4E, Supplementary Figure S8B). The Y1699C + G2019S combination also increased Ser1292 autophosphorylation *in vitro*, ∼12-fold, to a significantly greater extent than any other combination of mutations tested (Figure 4F, Supplementary Figure S8B).

### Activation of variants by overexpression of Rab29

**Figure 5.**
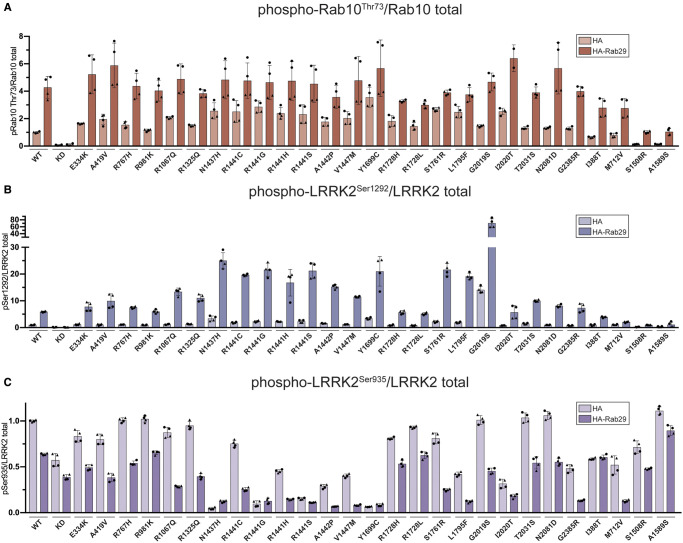
Selected LRRK2 variants are activated by Rab29. FLAG-tagged LRRK2 wildtype, kinase dead (KD = D2017A) and the indicated variants were transiently expressed in HEK293 cells with HA empty vector or HA-Rab29. Twenty-four hours post-transfection, cells were lysed and analysed by quantitative immunoblotting (as in Supplementary Figure S9). (**A**–**C**) Quantified immunoblotting data are presented as ratios of phospho-Rab10/total Rab10 (**A**), phospho-LRRK2 Ser1292/total LRRK2 (**B**), phospho-LRRK2 Ser935/total LRRK2 (**C**), normalized to the average of LRRK2 wildtype values for each replicate (mean ± SD). Combined immunoblotting data from two independent biological replicates (each performed in duplicate) are shown.

Overexpression of Rab29 recruits LRRK2 to the Golgi membrane, promoting stimulation of LRRK2 kinase activity as assessed by increased Rab10 phosphorylation and LRRK2 Ser1292 autophosphorylation [11,12,56]. We next studied the impact of Rab29 overexpression on the activating variants and observed enhanced pRab10^Thr73^ phosphorylation and Ser1292 autophosphorylation with all variants tested (Figure 5, Supplementary Figure S9). All ROC:COR_B_ domain interface variants enhanced Ser1292 autophosphorylation to a higher extent than the COR:COR interface variants (R1728H/L) following overexpression of Rab29 (Figure 5B, Supplementary Figure S9). Rab29 also increased the activity of the two variants displaying reduced activity (ROC (S1508R) and COR_A_ (A1589S)) (Figure 5A). Co-expression of Rab29 decreased LRRK2 Ser935 phosphorylation of most variants (Figure 5C). All LRRK2 variants phosphorylated Rab29 at Thr71 to a similar extent (Supplementary Figure S9), consistent with these being similarly activated by Rab29 binding.

### Impact of variants on MLi-2 induced microtubule association

**Figure 6.**
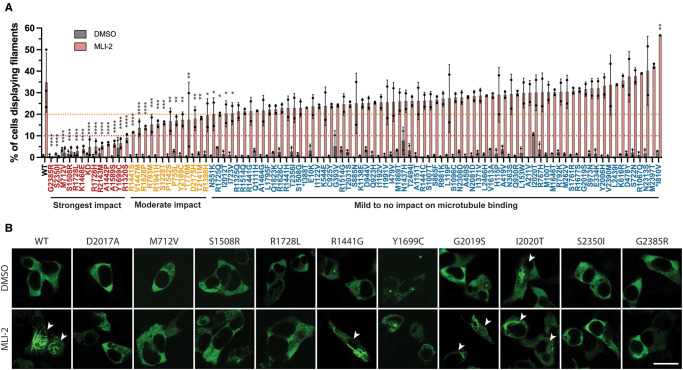
Impact of 98 LRRK2 variants on Type I inhibitor-induced microtubule association. (**A**) HEK293 cells transiently transfected with Flag-tagged LRRK2 wildtype, kinase dead (KD = D2017A) or the indicated variants were treated with 100 nM MLi-2 (or DMSO, control vehicle) for 3 h to induce microtubule association. Cells were then fixed and subjected to immunofluorescent microscopy imaging of Flag-tagged LRRK2. Data are presented as % of LRRK2 signal-positive cells that show filamentous LRRK2. Bars represent mean ± SD and each circle represents a data point from an independent experiment with at least 50 Flag-LRRK2 staining-positive cells evaluated. The full experiment with 98 variants was performed twice and select few variants with lower expression levels were tested again separately in a third smaller scale experiment. Two-way ANOVA with the Dunnett's multiple comparisons test was used to evaluate the statistical significance of the results (*P* values marked on the graph comparing the variant MLi-2-treated group to the WT MLi-2 treated group: *P < 0.05, **P < 0.01, ***P < 0.001, ****P < 0.0001. None of the DMSO treated groups showed statistically significant differences from the WT group). Data are arranged by % of cells with filamentous LRRK2 signal upon MLi-2 treatment (low to high). (**B**) Sample images of the Flag-LRRK2 staining of selected variants. Scale bar — 10 μm. Cells with filamentous LRRK2 are marked with white arrowheads.

As mentioned in the introduction, Type I LRRK2 inhibitors including MLi-2, promote ordered oligomerization of LRRK2 on filaments [24–27]. We next investigated how 98 of the 100 LRRK2 variants impacted MLi-2 induced microtubule association. Cells expressing wildtype or LRRK2 variants were treated ±100 nM MLi-2 for 3 h, prior to fixation with 4% (w/v) paraformaldehyde. Immunofluorescence analysis was performed blinded and the fraction of cells displaying filamentous LRRK2 was quantified by studying 50–221 LRRK2 signal-positive cells in two separate experiments (Figure 6A,B). For wild type and most of the variants analysed, in the absence of MLi-2, few of the cells, typically <5% displayed filamentous LRRK2. For a few variants (ARM (G393V, A419V), ROC (N1437H) COR_B_ (R1725Q), kinase (I2020T) and WD40 (T2494I)), moderately elevated filamentous LRRK2 was observed in the absence of MLi-2. Consistent with previous work, MLi-2 treatment markedly increased the proportion of cells displaying filamentous LRRK2 to above 20% for wildtype and most studied variants. On the contrary, the kinase-inactive LRRK2[D2017A] displayed no significant increase in filament formation following MLi-2 administration (Figure 6A,B). In addition, we observed that microtubule association in the presence of a Type I inhibitor was substantially reduced to below <10% of cells for 12 variants (ARM (M712V), LRR (R1320S), ROC (A1442P, K1468E, S1508R), COR_A_ (A1589S), COR_B_ (Y1699C, R1728H/L) and WD40 (R2143M, S2350I, G2385R)) (Figure 6A,B). We also observed that a further 13 variants (ANK (P755L, T776M, R793M), LRR (S1228T), ROC (R1398H, R1441S, V1447M), COR_A_ (P1542S, R1628P), kinase (R1941H) and WD40 (T2141M, D2175H, Y2189C)) displayed a moderate reduction in MLi-2 induced microtubule association (Figure 6A).

### Predicted impact of activating variants on LRRK2 structure

**Figure 7.**
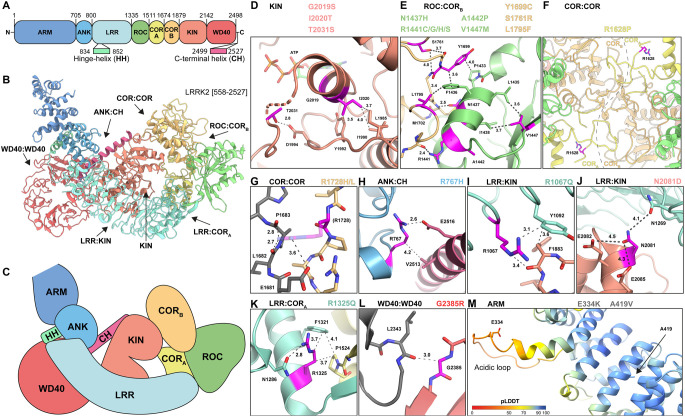
Structural analysis of identified activating LRRK2 variants. (**A**) Schematic domain overview of LRRK2 with domain boundaries. (**B**) Cartoon representation of LRRK2 [558–2527] with domains colored as in (**A**). Domain interfaces harboring activating mutations and kinase domain are indicated by black arrows. (**C**) Schematic representation of LRRK2 domains as viewed in (**B**). (**D**–**L**) Detailed views of LRRK2 variants in kinase active site (**D**), domain interfaces (**E**–**L**), coloring as in (**A**) and variants highlighted in magenta. Second LRRK2 molecule of dimer shown in gray (**G**,**L**). (**G**) R1728 side chain modeled in PyMOL shown as semi-transparent stick model. Distance measurements in Å are indicated by dark gray dashed lines. (**M**) Alphafold model of LRRK2 ARM domain colored by local confidence score (pLDDT) with variant residues shown as stick models. LRRK2 structures used are PDB 7LI4 (**B**,**D**,**E**,**H**–**K**), PDB 7LHT (**F**,**G**), PDB 6DLO (**L**), AFDB AF-Q5S007-F1_v1 (**M**).

The 23 identified activating variants are located across all domains of LRRK2 apart from the HH and C-terminal helix (Figure 3A). Utilizing available high-resolution structures of inactive full-length LRRK2 and LRRK2 WD40 domain dimer (Protein Data Bank (PDB) 7LI4, 7LHT [8], PDB 6DLO [36]), and the LRRK2 model from the EMBL-EBI AlphaFold database (AFDB) [57] (Figure 7A–C), we analyzed how these variants may impact LRRK2 structure and function. Three activating variants (G2019S, I2020T and T2031S) locate to the kinase active site (Figure 7D). Previous structural studies and molecular dynamics analysis of the G2019S and I2020T mutants suggest that they activate LRRK2 through changes in the flexibility of the kinase activation segment [8,58–60]. The T2031S variant is also located in the activation segment and may have a similar mechanism of kinase activation as G2019S and I2020T. An intriguing common feature of most of the other activating variants outside of the kinase domain is that they appear to destabilize LRRK2 interdomain interfaces. Most of these activating LRRK2 variants locate to the ROC and COR_B_ domains and lie within or nearby to the ROC:COR_B_ interface. These residues either participate in the interaction of the two domains directly (ROC (N1437H, R1441G/C/H/S), COR_B_ (Y1699C, L1795F)) or stabilize elements of the interface indirectly (ROC (A1442P, V1447M), COR_B_ (S1761R)) (Figure 7E). In addition, the COR_A_ variant R1628P and COR_B_ variants R1728H/L locate to the COR:COR dimer interface of LRRK2 (PDB 7LHT, Figure 7F,G). R1628 is located at the end of a loop in COR_A_ (residues 1613–1630) that interacts with the COR_B_ domain of the neighboring LRRK2 molecule, and the R1628P mutation may place this loop in an unfavorable conformation, hindering dimerization (Figure 7F). While the R1728 side chain is not fully resolved in the LRRK2 dimer structure, it is likely to form hydrogen bonds with the carbonyl-backbone of P1683 and L1682, as well as the side chain of E1681 (Figure 7G). The activating variants in the ROC and COR_B_ domains are, therefore, predicted to destabilize the ROC:COR_B_ and COR:COR interfaces. Furthermore, the R767H variant likely destabilizes the ANK:CH interface, as the arginine side chain of the R767 bridges the ANK domain to the C-terminal helix (CH) through hydrophobic and polar interactions with V2513 and E2516, respectively (Figure 7H). The LRR variant R167Q may disrupt the LRR:kinase interface, as R1067 interacts with the kinase N-lobe through polar interactions with the carbonyl-backbone of F1883 (Figure 7I). The CD-associated N2081D variant is also found in the LRR:kinase interface and forms a hydrophilic interaction with residues of the LRR domain (Figure 7J), and this mutation has been proposed to disrupt these interactions [8]. The R1325Q variant resides at the LRR:COR_A_ interface, where the aliphatic part of the arginine side chain is involved in hydrophobic contacts with F1321 and P1524, while the guanidinium group is engaged with N1286 via hydrophilic interactions (Figure 7K). The common G2385R risk factor variant maps to the WD40:WD40 dimer interface, and mutation of this residue to arginine is likely to cause steric clashes with the neighboring LRRK2 molecule and has been reported to block dimerization of this domain ([36], Figure 7L). Noteworthy, the G2385R variant may also exert its pathogenic effect through coulomb repulsion with R841 of the HH as previously proposed [8]. Finally, two of the identified activating variants, E334K and A419V, map to the N-terminal region of the ARM domain, which is absent from currently available high-resolution structures. However, the AlphaFold model of this region in LRRK2 (residues 159–511) has high local confidence scores (pLDDT) and agrees well with the experimentally determined cryo-EM map of LRRK2 (AFDB Q5S007-F1, EMD-23352, [8]) (Supplementary Figure S10). In the AlphaFold model, E334 locates to an unstructured loop (residues 328–347) that protrudes from the ARM repeats region (Figure 7M). Interestingly, this loop is highly acidic with 11 Asp/Glu out of 20 total residues and charge reversal through the E334K variant may, therefore, change the nature and function of this acidic loop. In contrast, the second ARM variant A419V is buried in the ARM repeats and is not solvent exposed, and mutation to Val is likely to disturb the ARM structure (Figure 7M). Together, the activating ARM variants point to a third mechanism in regulating LRRK2 kinase activity, not yet explained by existing structures. We speculate that these mutations may affect substrate access, as the N-terminal ARM has previously been implicated in Rab substrate binding [13].

### Exploring the mechanism of LRRK2 activation by disruption of interdomain interfaces

**Figure 8.**
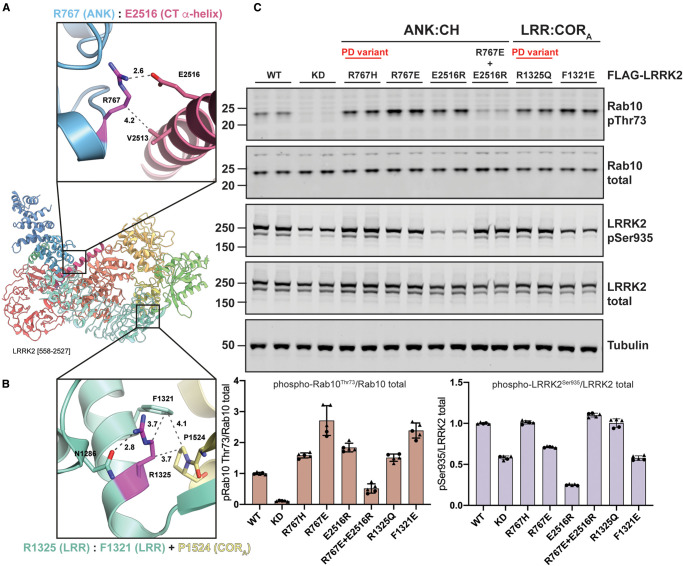
Structure-guided mutations in the N-terminus of LRRK2 stimulate LRRK2-mediated Rab10 phosphorylation. (**A**,**B**) Cartoon representation of LRRK2 [558–2527] with detailed views of ANK (blue):CT α-helix (magenta) (**A**) and LRR (green):COR_A_ (yellow) (**B**) interactions. Distance measurements in Å are indicated by dark gray dashed lines. (Right panel) HEK293 cells were transfected with wildtype, kinase dead (KD = D2017A), and the indicated LRRK2 variants. Each lane represents a different dish of cells. Cells were harvested 24 h post-transfection and subjected to quantitative immunoblot analysis with the indicated antibodies. Each lane represents a different dish of cells. The ratios of phospho-Rab10 Thr73/total Rab10 and phospho-LRRK2 Ser935/total LRRK2 were normalized to wildtype LRRK2 values. Quantified data are presented as mean ± SD and are representative of two independent experiments.

A common theme emerging in our analysis of the identified activating variants is that the apparent disruption of interdomain interfaces leads to activation of LRRK2. We, therefore, decided to explore this hypothesis by introducing structure-guided mutations in the ANK:CH and LRR:COR_A_ interfaces. As outlined above, the two N-terminal, PD-associated variants namely R767H (ANK, domain, Figure 8A) and R1325Q (LRR domain, Figure 8B), activate LRRK2. Structural analysis indicates that the R767H mutation would disrupt an ionic interaction with E2516 residue located within the long C-terminal alpha-helix domain. Consistent with this, we find that the E2516R mutation (not a PD-associated variant) activates LRRK2 (Figure 8C). Reinstating this ionic interaction by generating charge reversed, double R767E and E2516R mutations restores LRRK2 activity to that of wildtype enzyme (Figure 8C). Similarly, the R1325Q activating variant in the LRR domain is predicted to disrupt a hydrophobic interaction with F1321 (LRR) and P1524 (COR_A_) (Figure 8B). Consistent with this, disrupting this hydrophobic network by generating a F1231E mutation is sufficient to activate LRRK2 (Figure 8C). Together with the structural analysis of activating PD variants, these experiments strengthen the hypothesis that a disruption of LRRK2 interdomain interfaces can cause an increase in LRRK2 kinase activity.

## Discussion

**Figure 9.**
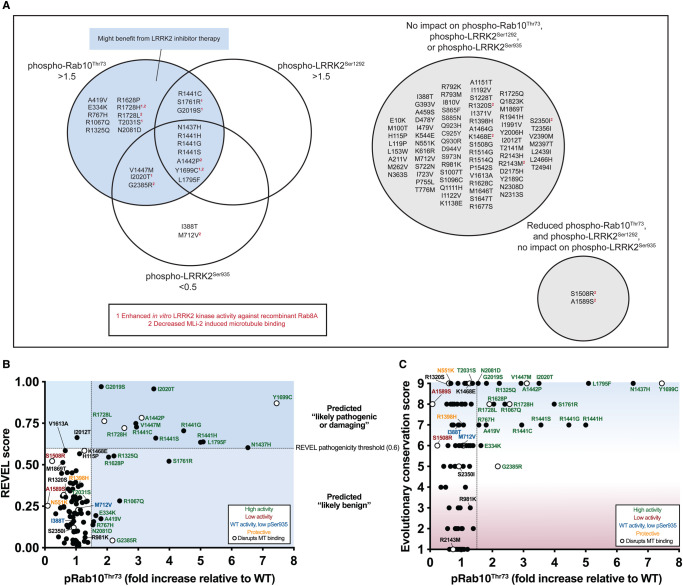
Correlation between activating LRRK2 mutations and high REVEL pathogenicity prediction or high evolutionary amino acid conservation scores. (A) Schematic summarizing biochemical data of 100 LRRK2 variants and categorization of variants based on Rab10 phosphorylation, LRRK2 Ser1292 phosphorylation, and biomarker phosphorylation. Variants that enhance *in vitro* LRRK2 kinase activity or block MLi-2 induced microtubule binding are marked with a superscript highlighted in red. (B) REVEL scores for LRRK2 variants were acquired from Bryant et al. [41] or through the online pathogenicity prediction tool http://database.liulab.science/dbNSFP. REVEL scores were plotted against phospho-Rab10/total Rab10 ratios acquired for each LRRK2 variant that were normalized to wildtype. High activity variants (pRab10^Thr73^ > 1.5-fold relative to wildtype) are marked in green, low activity variants (similar to kinase-inactive LRRK2) are marked in red, protective variants are marked in yellow, and variants that block MLi-2 induced microtubule binding are represented with an open circle. The REVEL pathogenicity threshold is marked with a dashed line (0.6). Above this line are variants predicted to be ‘likely pathogenic or damaging,' and variants below this line are predicted to be ‘likely benign.' (C) LRRK2 orthologue sequences were acquired from OrthoDB. Orthologue sequences were aligned using MAFFT. The multiple sequence alignment of LRRK2 orthologues was submitted to the ConSurf server to determine evolutionary conservation scores for LRRK2 amino acids (1 is low conservation and 9 is high conservation). Conservation scores were plotted against pRab10^Thr73^/total Rab10 ratios acquired for each LRRK2 variant that were normalized to wildtype. Activating variants (pRab10^Thr73^ > 1.5-fold relative to wildtype) are marked in green, low activity variants (similar to kinase-inactive LRRK2) are marked in red, protective variants are marked in yellow, and variants that block MLi-2 induced microtubule binding are represented with an open circle.

In this study, we employed a tried and tested workflow to assess the activity of 100 LRRK2 variants that have previously been linked in the literature to PD (Supplementary Table S1). The data obtained with respect to how each variant impacts LRRK2-mediated phosphorylation of Rab10 at Thr73, LRRK2 biomarker phosphorylation at Ser935, and LRRK2 autophosphorylation at Ser1292 are summarized in Figure 9A. Most importantly, we have characterized a group of 23 variants that reproducibly and robustly stimulate LRRK2-mediated pRab10^Thr73^ substrate phosphorylation >1.5-fold in the HEK293 cell overexpression system (Figures 1–3). We investigated how the computational pathogenicity REVEL score [41] of each variant (Supplementary Table S1) correlates with experimentally measured variant activity and observed that 13 of the 23 activating variants possessed a REVEL score of >0.6, which is considered pathogenic (Figure 9B). These include most of the previously characterized pathogenic mutations, as well as some of the novel variants identified in this study (ROC (A1442P, V1447M), COR_B_ (L1795F, R1728L)). The activating variants located outside of the catalytic domains (ARM (E334K, A419V), ANK (R767H), LRR (R1067Q, R1325Q)) displayed REVEL scores of <0.6 suggesting that this algorithm may be less effective at predicting activating variants lying outside the catalytic domains (Figure 9B). Only one of the variants tested, I2012T, possessed a REVEL score >0.6 and did not stimulate LRRK2 in our assays (Figure 9B). 74 of the 77 that did not enhance LRRK2 activity, displayed a REVEL score of <0.6 (Figure 9B). We next explored how the evolutionary conservation score of each variant residue (Supplementary Table S1) correlates with cellular kinase activity (Figure 9C). For this analysis, the evolutionary conservation was calculated using the ConSurf Server [46] and given a score of 1–9, with 1 being the least conserved, 4 being average and 9 being the most conserved. Functionally important residues that play an important role in controlling kinase activity would be expected to be highly evolutionarily conserved. Consistent with this, 21 of the 23 LRRK2 activating variants possess a high evolutionary conservation score of 7, 8, or 9 (Figure 9C). Only two activating variants, namely E334K (score 6) and G2385R (score 5), possess a lower score. Interestingly, the LRR R981K variant that was selected for our secondary screen and found not to significantly increase LRRK2 activity (Figure 3), possessed a low REVEL (0.062) and conservation score (3), consistent with these parameters having utility in predicting pathogenicity.

Our data suggest that the impact of variants on LRRK2 kinase activity is better assessed employing a cellular assay measuring LRRK2-dependent pRab10^Thr73^ levels (Figures 1–3), rather than an immunoprecipitation *in vitro* kinase assay (Figure 4), as only 7 of the 22 variants analysed, both enhanced activity in the cellular assay and stimulated LRRK2 kinase activity *in vitro* (COR_B_ (Y1699C, R1728H, R1728L, S1761R) and kinase (G2019S, I2020T and T2031S)). It is likely that the mutants that activate LRRK2 in the *in vitro* assay, stabilize the active conformation of the kinase domain. Further work is required to understand the mechanism by which most of the other identified variants stimulate LRRK2 kinase activity in cells. It is possible that these variants facilitate membrane interaction and/or association with other factors that enhance LRRK2-mediated phosphorylation of pRab10^Thr73^. In future work, it would be interesting to explore whether the Cys residue in the Y1699C variant becomes oxidized or forms a disulfide bond and whether this could contribute to cellular and/or *in vitro* activation observed with this variant. We also observed that combinations of Y1699C with G2019S or T2031S stimulated *in vitro* LRRK2 kinase activity to a greater extent than each individual variant alone (Figure 4E) and this finding may be useful in generating more active LRRK2 constructs for future functional and/or structural analysis. The finding that three variants, namely A1442P (ROC), T2031S (kinase) and N2081D (kinase), enhanced Rab10 phosphorylation without impacting Rab12 phosphorylation (Figure 3) indicates that certain variants could differentially impact the phosphorylation of Rab proteins. It would be interesting to explore this further, and this could be done using a recently described multiplexed mass spectrometry assay [61].

The majority of the 23 variants that stimulate LRRK2 activity reduce biomarker site phosphorylation. This is consistent with previous work and has been interpreted to imply that active variants might adopt a conformation in which the biomarker sites are poorly phosphorylated or more efficiently dephosphorylated by the upstream kinase or phosphatase that acts on these sites [29,53]. Consistent with this, Type I inhibitors that stabilize the active conformation of LRRK2 induce dephosphorylation of these biomarker sites [28]. Type II inhibitors that stabilize the inactive conformation of LRRK2 do not induce dephosphorylation of these residues [30]. However, our results emphasize that several activating variants located within the ANK (R767H) or kinase (G2019S, T2031S and N2081D) domains do not significantly reduce the phosphorylation of Ser935 or other biomarker sites (Figures 2B and 3). The reasons for this are currently not known. Perhaps these variants when expressed in cells retain a conformation distinct from the other activating variants. All activating variants within the ROC, COR_A_ or COR_B_ domains display reduced biomarker site phosphorylation. Our data also emphasize that autophosphorylation at Ser1292 is not directly correlated with LRRK2 kinase activity toward Rab substrates. LRRK2 G2019S shows by far the highest levels of Ser1292 phosphorylation among the pathogenic mutants, while its activity towards Rab10 is lower than other LRRK2 pathogenic mutants (Figures 1C, 2A and 3).

We also identify 12 variants (ARM (M712V), LRR (R1320S), ROC (A1442P, K1468E, S1508R), COR_A_ (A1589S), COR_B_ (Y1699C, R1728H/L) and WD40 (R2143M, S2350I, G2385R)), in addition to kinase-inactive LRRK2[D2017A], that significantly suppressed microtubule association in the presence of the MLi-2 Type I LRRK2 kinase inhibitor (Figure 6). Previous studies have established that the ROC-COR-kinase-WD40 domain fragment is sufficient to mediate oligomerization onto microtubules [9], and revealed that mutations impacting the WD40:WD40 interface [24] or the COR:COR interface [8], block microtubule association in cells. These conclusions are confirmed by a recent study that also highlights that disrupting the COR:COR interface by introducing the R1731L/D mutation, or the WD40:WD40 interface by introducing the S2343D mutation, markedly blocks microtubule association [10]. These findings likely account for why we observed that the COR_B_ (R1728H/R1728L), as well as the WD40 (R2143M, S2350I, G2385R) variants that likely affect the COR:COR and WD40:WD40 interfaces, inhibit microtubule association. Recent analysis identified a set of key basic residues located within the ROC domain that directly interact with acidic microtubule residues and this interaction is disrupted by a ROC (R1501W) variant linked to PD [10]. The ROC S1508R variant that we identified to block microtubule association is an internally buried residue in the ROC domain that is located adjacent to the basic microtubule-interacting patch and could affect the positioning of the basic microtubule-binding patch. K1468E is on the ROC domain surface pointing towards the microtubule surface. This residue is not part of the characterized microtubule-binding basic patch but located nearby and could also participate in microtubule binding. If the kinase-inactivating D2017A variant impacted MLi-2 binding, this would account for why this mutation blocks MLi-2 mediated microtubule association. A previous study has also noted that a kinase-inactivating mutation distinct to that used in this study, namely K1906R, also blocked LRRK2 from association with microtubules [26]. The mechanism by which ARM (M712V), LRR (R1320S), ROC (A1442P) and COR_B_ (Y1699C) variants interfere with microtubule binding is currently not clear and requires further investigation.

It has been suggested that the ability of pathogenic variants to associate with microtubules may be linked to PD [9]. The finding that seven activating variants (ROC (A1442P), COR_B_ (Y1699C, R1728H/L) and WD40 (R2143M, S2350I, G2385R)) displayed reduced microtubule association, highlights that further analysis is required to explore a potential link between microtubule binding and PD. Interestingly, a recent study characterizing the R1731L and R1731D mutations that interfere with the COR:COR dimer interface, also observed that these mutations enhanced LRRK2 kinase activity [10], similar to what we have observed with the R1728H/L variants. The mechanism by which disruption of the COR:COR interface promotes kinase activation is currently unknown. It should be noted that pathogenic Flag-tagged LRRK2 mutants display minimal filament formation in the absence of MLi-2 (Figure 6), which is in contrast with previous reports employing GFP-tagged pathogenic LRRK2 mutants [24–26]. It would be worth investigating whether the known properties of certain GFP variants [62] to dimerize/oligomerize could account for the increased microtubule binding observed in other studies.

LRRK2 is a large protein with 2527 residues; over 1000 variants have been reported thus far and many people with PD likely carry additional rare LRRK2 variants of unknown clinical significance [41]. With next-generation sequencing becoming more readily available for people with PD and disease modification with targeted treatments including LRRK2 kinase inhibitors entering clinical trials [63,64], there is increased urgency to have a concrete set of functional parameters and experimental workflows available to help clinicians assess whether any given LRRK2 variant is likely to increase kinase activity and therefore a likely driver of the disease. We advocate the use of the experimental HEK293 cellular assay (Figures 1–3) to assess whether a variant enhances LRRK2 kinase activity by measuring LRRK2-dependent pRab10^Thr73^ phosphorylation, rather than the *in vitro* kinase assay (Figure 4) or solely relying on predictive methodology. Although the REVEL pathogenic score is useful, our data highlight that the REVEL score fails to identify most activating variants lying outside the GTPase and kinase domains (Figure 9B). A conservation evolutionary score of 7–9 would also indicate that the variant lies within a functionally critical position of the protein and most activating variants displayed conservation scores within this range. In future work, we are planning to experimentally assess additional LRRK2 variants linked to PD as they are reported. We are aiming to deposit all experimental data, REVEL and evolutionary conservation scores into a publicly accessible database (e.g. in collaboration with the Movement Disorder Society Genetic mutation database (www.MDSGene.org)) as soon as data become available. We hope that this information will help clinicians interpret LRRK2 variants in terms of their pathogenicity for genetic counseling, stratify people with LRRK2 variants and make a case to prioritize these individuals for LRRK2-targeting clinical trials, as well as stimulate further mechanistic and structural analysis to better understand how these variants enhance LRRK2 kinase activity. There is also a need to validate similar functional workflows for other PD-associated genes, including the lysosomal enzyme glucocerebrosidase (GBA1) [65].

## Materials and methods

### Reagents

MLi-2 LRRK2 inhibitor was synthesized by Natalia Shpiro (University of Dundee). Human recombinant Rab8A (1–207, DU47363) used for the immunoprecipitation kinase assays was obtained from the MRC PPU Reagents and Services (https://mrcppureagents.dundee.ac.uk).

#### Cell culture

HEK293 cells (ATCC Cat no. CRL-1573, RRID:CVCL_0045) were cultured in DMEM (Dulbecco's Modified Eagle Medium) (Dulbecco's Modified Eagle Medium, Gibco™) containing 10% (v/v) fetal calf serum,  mM l-glutamine, 100 U/ml penicillin and 100 μg/ml streptomycin at 37°C in a humidified incubator maintaining 5% (v/v) CO_2_. Cells were regularly tested for mycoplasma contamination.

#### Plasmids

All plasmids used in this study were obtained from the MRC PPU Reagents and Services (https://mrcppureagents.dundee.ac.uk) and these are listed in Table 1. Each LRRK2 variant was confirmed by sequencing at the MRC Sequencing and Services (https://www.dnaseq.co.uk) and the amplified plasmid preparation quality was validated via agarose gel electrophoresis using ethidium bromide staining. All plasmids are available to request via the MRC PPU Reagents and Services website (https://mrcppureagents.dundee.ac.uk).

**Table 1.** List of plasmids used in this study

DU number	Construct	Plasmid
DU44060	Flag-empty	pCMV5D
DU6841	Flag LRRK2 wildtype	pCMV5
DU10128	Flag LRRK2 D2017A (KD)	pCMV5
DU13826	Flag LRRK2 E10K	pCMV5
DU62019	Flag LRRK2 M100T	pCMV5
DU68340	Flag LRRK2 H115P	pCMV5
DU68326	Flag LRRK2 L119P	pCMV5
DU62020	Flag LRRK2 L153W	pCMV5
DU26913	Flag LRRK2 A211V	pCMV5
DU68327	Flag LRRK2 M262V	pCMV5
DU26914	Flag LRRK2 E334K	pCMV5
DU26911	Flag LRRK2 N363S	pCMV5
DU68328	Flag LRRK2 I388T	pCMV5
DU68329	Flag LRRK2 G393V	pCMV5
DU26842	Flag LRRK2 A419V	pCMV5
DU62596	Flag LRRK2 A459S	pCMV5
DU68330	Flag LRRK2 D478Y	pCMV5
DU68331	Flag LRRK2 I479V	pCMV5
DU13049	Flag LRRK2 K544E	pCMV5
DU26736	Flag LRRK2 N551K	pCMV5
DU62834	Flag LRRK2 K616R	pCMV5
DU26915	Flag LRRK2 M712V	pCMV5
DU62002	Flag LRRK2 S722N	pCMV5
DU68332	Flag LRRK2 I723V	pCMV5
DU26926	Flag LRRK2 P755L	pCMV5
DU26708	Flag LRRK2 R767H	pCMV5
DU72489	Flag LRRK2 R767E	pCMV5
DU68333	Flag LRRK2 T776M	pCMV5
DU62016	Flag LRRK2 R792K	pCMV5
DU26912	Flag LRRK2 R793M	pCMV5
DU26907	Flag LRRK2 I810V	pCMV5
DU26722	Flag LRRK2 S865F	pCMV5
DU26709	Flag LRRK2 S885N	pCMV5
DU62855	Flag LRRK2 Q923H	pCMV5
DU62012	Flag LRRK2 C925Y	pCMV5
DU13164	Flag LRRK2 Q930R	pCMV5
DU68334	Flag LRRK2 D944V	pCMV5
DU13082	Flag LRRK2 S973N	pCMV5
DU62038	Flag LRRK2 R981K	pCMV5
DU62003	Flag LRRK2 S1007T	pCMV5
DU13043	Flag LRRK2 R1067Q	pCMV
DU13044	Flag LRRK2 S1096C	pCMV
DU13988	Flag LRRK2 Q1111H	pCMV
DU13286	Flag LRRK2 I1122V	pCMV5
DU26724	Flag LRRK2 K1138E	pCMV5
DU19010	Flag LRRK2 A1151T	pCMV
DU17133	Flag LRRK2 I1192V	pCMV
DU13045	Flag LRRK2 S1228T	pCMV
DU62001	Flag LRRK2 R1320S	pCMV5
DU72476	Flag LRRK2 F1321E	pCMV5
DU62011	Flag LRRK2 R1325Q	pCMV5
DU13046	Flag LRRK2 I1371V	pCMV5
DU26565	Flag LRRK2 R1398H	pCMV5
DU26643	Flag LRRK2 N1437H	pCMV5
DU13078	Flag LRRK2 R1441C	pCMV5
DU13077	Flag LRRK2 R1441G	pCMV
DU13287	Flag LRRK2 R1441H	pCMV
DU62906	Flag LRRK2 R1441S	pCMV5
DU13170	Flag LRRK2 A1442P	pCMV5
DU62501	Flag LRRK2 V1447M	pCMV5
DU62849	Flag LRRK2 A1464G	pCMV5
DU68324	Flag LRRK2 K1468E	pCMV5
DU62848	Flag LRRK2 S1508R	pCMV5
DU62836	Flag LRRK2 S1508G	pCMV5
DU67599	Flag LRRK2 R1514G	pCMV5
DU13047	Flag LRRK2 R1514Q	pCMV5
DU68335	Flag LRRK2 P1542S	pCMV5
DU68336	Flag LRRK2 A1589S	pCMV5
DU19019	Flag LRRK2 V1613A	pCMV5
DU68325	Flag LRRK2 R1628C	pCMV5
DU19007	Flag LRRK2 R1628P	pCMV5
DU26840	Flag LRRK2 M1646T	pCMV5
DU62804	Flag LRRK2 T1647S	pCMV5
DU62517	Flag LRRK2 R1677S	pCMV5
DU26486	Flag LRRK2 Y1699C	pCMV5
DU62840	Flag LRRK2 R1725Q	pCMV5
DU17138	Flag LRRK2 R1728H	pCMV5
DU17139	Flag LRRK2 R1728L	pCMV5
DU62839	Flag LRRK2 S1761R	pCMV5
DU17134	Flag LRRK2 L1795F	pCMV5
DU62838	Flag LRRK2 Q1823K	pCMV5
DU13169	Flag LRRK2 M1869T	pCMV5
DU13079	Flag LRRK2 R1941H	pCMV5
DU62837	Flag LRRK2 I1991V	pCMV5
DU13880	Flag LRRK2 Y2006H	pCMV5
DU13080	Flag LRRK2 I2012T	pCMV5
DU10129	Flag LRRK2 G2019S	pCMV5
DU13081	Flag LRRK2 I2020T	pCMV5
DU17135	Flag LRRK2 T2031S	pCMV5
DU26721	Flag LRRK2 N2081D	pCMV5
DU17140	Flag LRRK2 T2141M	pCMV5
DU72441	Flag LRRK2 R2143H	pCMV5
DU17141	Flag LRRK2 R2143M	pCMV5
DU62374	Flag LRRK2 D2175H	pCMV5
DU62391	Flag LRRK2 Y2189C	pCMV5
DU62004	Flag LRRK2 N2308D	pCMV5
DU62005	Flag LRRK2 N2313S	pCMV5
DU62502	Flag LRRK2 S2350I	pCMV5
DU62375	Flag LRRK2 T2356I	pCMV5
DU27381	Flag LRRK2 G2385R	pCMV5
DU62376	Flag LRRK2 V2390M	pCMV5
DU26735	Flag LRRK2 M2397T	pCMV5
DU62393	Flag LRRK2 L2439I	pCMV5
DU17142	Flag LRRK2 L2466H	pCMV5
DU30901	Flag LRRK2 T2494I	pCMV5
DU72475	Flag LRRK2 E2516R	pCMV5
DU62832	Flag LRRK2 R1441G + T1647S	pCMV5
DU62805	Flag LRRK2 G2019S + T1647S	pCMV5
DU68883	Flag LRRK2 Y1699C + G2019S	pCMV5
DU68846	Flag LRRK2 Y1699C + T2031S	pCMV5
DU26749	Flag LRRK2 R1398H + R1441G	pCMV5
DU26704	Flag LRRK2 R1398H + Y1699C	pCMV5
DU26703	Flag LRRK2 R1398H + G2019S	pCMV5
DU72659	Flag LRRK2 R767E + E2516R	pCMV5
DU49303	HA-empty	pCMV5D
DU50222	HA-Rab29	pCMV5D

#### Cell transfection and lysis

A protocols.io description of our cell transfection (dx.doi.org/10.17504/protocols.io.bw4bpgsn) and cell lysis method (dx.doi.org/10.17504/protocols.io.b5jhq4j6) has previously been described. For LRRK2 variant immunoblot analysis, HEK293 cells were seeded into six-well plates and transiently transfected at 60–70% confluence using polyethylenimine (PEI) transfection reagent with Flag-empty, Flag-LRRK2 wildtype or variant plasmids. Two micrograms of plasmid and 6 µg of PEI were diluted in 0.5 ml of Opti-MEM™ Reduced serum medium (Gibco™) per single well. Cells were lysed 24 h post-transfection in an ice-cold lysis buffer containing 50 mM Tris–HCl pH 7.4, 1 mM EGTA, 10 mM 2-glycerophosphate, 50 mM sodium fluoride, 5 mM sodium pyrophosphate, 270 mM sucrose, supplemented with 1 µg/ml microcystin-LR, 1 mM sodium orthovanadate, complete EDTA-free protease inhibitor cocktail (Roche), and 1% (v/v) Triton X-100. Lysates were clarified by centrifugation at 15 000 *g* at 4°C for 15 min and supernatants were quantified by Bradford assay.

For LRRK2 variant screen for microtubule association, cells were split into either µ-Plate 24-wells (no. 1.5 polymer coverslip, black well, flat bottom, ibiTreat; Ibidi) for immunofluorescence or regular 24-well plates for immunoblotting control. Cells were transfected using PEI transfection with Flag-empty, Flag-LRRK2 wildtype or variant plasmids; 0.6 µg of plasmid and 1.7 µg of PEI in 0.15 ml of Opti-MEM™ per single well. Three hours prior to lysis, cells were treated with 100 nM MLi-2 or 0.1% (v/v) DMSO (vehicle). Forty-eight hours post-transfection, cells for immunofluorescence were fixed for 10 min using 4% (v/v) PFA in PBS (phosphate buffered saline), pre-warmed to 37°C and cells for immunoblotting were lysed as above.

#### Quantitative immunoblot analysis

A protocols.io description of our quantitative immunoblotting protocol has previously been described (dx.doi.org/10.17504/protocols.io.bsgrnbv6). Briefly extracts were mixed with a quarter of a volume of 4× SDS–PAGE loading buffer (250 mM Tris–HCl, pH 6.8, 8% (w/v) SDS, 40% (v/v) glycerol, 0.02% (w/v) bromophenol blue and 5% (v/v) 2-mercaptoethanol) and heated at 95°C for 5 min. Samples were loaded onto NuPAGE 4–12% Bis–Tris Midi Gels (Thermo Fisher Scientific, Cat no. WG1402BOX or Cat no. WG1403BOX) or self-cast 10% Bis–Tris gels and electrophoresed at 130 V for 2 h with NuPAGE MOPS SDS running buffer (Thermo Fisher Scientific, Cat no. NP0001-02). At the end of electrophoresis, proteins were electrophoretically transferred onto a nitrocellulose membrane (GE Healthcare, Amersham Protran Supported 0.45 µm NC) at 90 V for 90 min on ice in transfer buffer (48 mM Tris base and 39 mM glycine supplemented with 20% (v/v) methanol). The membranes were blocked with 5% (w/v) skim milk powder dissolved in TBS-T (50 mM Tris base, 150 mM sodium chloride (NaCl), 0.1% (v/v) Tween 20) at room temperature for 1 h. Membranes were washed three times with TBS-T and were incubated in primary antibody overnight at 4°C. Prior to secondary antibody incubation, membranes were washed three times for 15 min each with TBS-T. The membranes were incubated with secondary antibody for 1 h at room temperature. Thereafter, membranes were washed with TBS-T three times with a 15 min incubation for each wash, and protein bands were acquired via near-infrared fluorescent detection using the Odyssey CLx imaging system and quantified using Image Studio Lite (Version 5.2.5, RRID:SCR_013715).

**Table 2.** List of antibodies used in this study

Antibody target	Company	Cataloge number (RRID)	Host species	Dilution
LRRK2 (C-terminus)	Antibodies Incorporated/NeuroMab	75–253 (RRID:AB_10675136)	Mouse	1:1000
LRRK2 pSer935	MRC PPU Reagents and Services, University of Dundee	UDD2 10(12) (RRID:AB_2921228)	Rabbit	1 µg/ml
LRRK2 pSer955	Abcam Inc.	ab169521 (RRID:AB_2921221)	Rabbit	1:1000
LRRK2 pSer973	Abcam Inc.	ab181364 (RRID:AB_2921222)	Rabbit	1:1000
LRRK2 pSer1292	Abcam Inc.	ab203181 (RRID:AB_2921223)	Rabbit	1:2000
LRRK2 pThr1357	Abcam Inc.	ab270606 (RRID:AB_2921224)	Rabbit	1:1000
LRRK2 pThr1503	Abcam Inc.	ab154423 (RRID:AB_2921225)	Rabbit	1:1000
Rab8A pThr72	Abcam Inc.	ab230260 (RRID:AB_2814988)	Rabbit	1:1000
Rab8A	Sigma–Aldrich	WH0004218M2 (RRID:AB_1843239)	Mouse	1 µg/ml
Rab10 pThr73	Abcam Inc.	ab230261 (RRID:AB_2811274)	Rabbit	1:1000
Rab10	Nanotools	0680–100/Rab10-605B11 (RRID:AB_2921226)	Mouse	1:500
Rab12 pSer106	Abcam Inc.	ab256487 (RRID:AB_2884880)	Rabbit	1:1000
Rab12	MRC PPU Reagents and Services, University of Dundee	SA227 (RRID:AB_2921227)	Sheep	1 µg/ml
Rab29 pThr71	Abcam Inc.	ab241062 (RRID:AB_2884878)	Rabbit	1:1000
HA	Sigma–Aldrich	11867423001 (RRID:AB_390918)	Rat	1:1000
Alpha-tubulin	Cell Signalling Technologies	3873S (RRID:AB_1904178)	Mouse	1:1000
Secondary antibodies	Company	Cataloge number (RRID)	Dilution	
IRDye 800CW Donkey anti-Rabbit IgG	LI-COR	926–32213 (RRID:AB_621848)	1:10 000	
IRDye 800CW Goat-anti-Rabbit IgG	LI-COR	926–32 211 (RRID:AB_621843)	1:10 000	
IRDye 680LT Donkey anti-Mouse IgG	LI-COR	926–68 022 (RRID:AB_10715072)	1:10 000	
IRDye 800CW Donkey anti-Mouse IgG	LI-COR	926–32 212 (RRID:AB_621847)	1:10 000	
IRDye 680LT Goat-anti-Rat IgG	LI-COR	926–68 029 (RRID:AB_10715073)	1:10 000	
IRDye 680LT Donkey anti-Goat IgG	LI-COR	926–68 024 (RRID:AB_10706168)	1:10 000	

#### Antibodies

A list of antibodies employed in this study is presented in Table 2.

#### Immunoprecipitation kinase assays

A protocols.io description of our LRRK2 immunoprecipitation kinase assay has previously been described (dx.doi.org/10.17504/protocols.io.bw4bpgsn). Briefly, HEK293 cells were transiently transfected with FLAG-LRRK2 wildtype, FLAG-LRRK2 D2017A and FLAG-tagged LRRK2 variants using PEI and lysed 24 h post-transfection. Prior to immunoprecipitation, cell lysates were subjected to quantitative immunoblotting to assess the expression of each LRRK2 variant by quantifying total LRRK2 and normalizing to Tubulin. One hundred micrograms cell lysate expressing FLAG-LRRK2 wild-type, and the equivalent amount of cell lysate adjusted according to the expression of each FLAG-tagged LRRK2 variant, was used to immunoprecipitate LRRK2 with 10 µl anti-FLAG M2 resin for 1 h at 4°C, and immunoprecipitations were set up in triplicate per dish of cells. Immunoprecipitates were washed three times with lysis buffer supplemented with 300 mM NaCl, and twice with 50 mM Tris–HCl (pH 7.5). Kinase reactions were set up in a total volume of 25 µl, with immunoprecipitated LRRK2 in 50 mM Tris–HCl (pH 7.5), 10 mM MgCl_2_, 1 mM ATP, in the presence of 5 µg recombinant Rab8A. Kinase reactions were carried out at 30°C for 45 min at 1150 rpm. Reactions were terminated by adding 25 µl 2× LDS (lithium dodecyl sulfate) loading buffer to the beads. After heating the reactions at 70°C for 15 min, the eluates were collected by centrifugation through a 0.22 µM pore-size Spin-X column and supplemented with 2% (v/v) 2-mercaptoethanol. The kinase reactions were heated at 95°C for 5 mins, then subjected to quantitative immunoblot analysis. Membranes were developed using the Licor Odyssey CLx scan Western Blot imaging system and quantified using Image Studio Lite (Version 5.2.5, RRID:SCR_013715).

#### Immunofluorescence, imaging, and cell counting and quantitation of microtubule binding

A protocols.io description of the Immunofluorescence-based method that we used to assess LRRK2 association with microtubules in HEK293 cells has been described (dx.doi.org/10.17504/protocols.io.b5jhq4j6). Cells were fixed in 4% (w/v) paraformaldehyde (Sigma–Aldrich no. P6148) in PBS, pH 7.4 for 10 min, followed by permeabilization using 1% (v/v) NP-40 in PBS for 10 min. Cells were then blocked in 1% (w/v) bovine serum albumin in PBS for 1 h at room temperature. Blocked cells were incubated with Flag M2 (raised in mouse, Sigma–Aldrich Cat no. F1804, RRID:AB_262044, 1:1000 dilution) and β-tubulin (raised in rabbit, Abcam Cat no. ab6046, RRID:AB_2210370, 1:500 dilution) antibodies for 2 h at 37°C. Cells were washed 3 times (15 min each) with 0.2% (w/v) bovine serum albumin in PBS and incubated with secondary antibodies (goat-anti-mouse Alexa Fluor 488 Thermo Fisher Scientific Cat no. A-21202, RRID:AB_141607 and goat-anti-rabbit Alexa Fluor 594 Thermo Fisher Scientific Cat no. A-21207, RRID:AB_141637, 1:500 dilution) and 1 µg/ml DAPI (4′,6-diamidino-2-phenylindole, Dilactate) for 1 h at room temperature in the dark. Cells were washed 3 times (15 min each) with 0.2% (w/v) bovine serum albumin in PBS and were kept in PBS at 4°C until imaging. Plates were imaged on the Zeiss LSM 710 or 880 laser scanning microscopes using the ×40 EC Plan-Neofluar (NA 1.3) objective with a zoom of 0.6 and optical section thickness of 1.0 µm (image size 2048 × 2048 pixels, pixel size 0.173 mm). Four to six randomly selected fields with Alexa Fluor 488(FLAG)-positive cells were collected for each well blinded to LRRK2 variant and treatment conditions. For further cell counting, LRRK2 variant and treatment condition were blinded from the counter by renaming the image files using a simple Python code script (IPython (RRID:SCR_001658). The Python code script used to rename image files for blinded analysis of immunofluorescence images was deposited to Zenodo via GitHub: doi:10.5281/zenodo.6801448. Cells containing any filamentous shapes of the Alexa Fluor 488 signal were counted as ‘filamentous,' ones without filamentous signal but with punctate staining were counted as ‘punctate,' and the remaining cells with fully cytosolic signal were counted as ‘cytosolic.' DAPI and β-tubulin signal was used to make sure only cells containing a single nucleus were counted, avoiding cells that have not finished dividing or are multi-nuclear. Variants with a statistically significant and largest effect on inhibitor-induced LRRK2 filament formation (<10% of LRRK2 signal-positive cells after MLi-2 treatment compared with the 34.7% of MLi-2 treated wildtype LRRK2 cells) were labeled as the ‘Strongest impact' group, the remaining variants with a statistically significant decrease in LRRK2 filament formation (<21%) were labeled as ‘Moderate impact' and the remaining variants were labeled as ‘Microtubule binding not significantly impacted'.

#### Immunoblotting data analysis

Immunoblotting data (acquired using a LI-COR CLx Western Blot imaging system) were quantified using Image Studio Lite (Version 5.2.5, RRID:SCR_013715). Quantified data were plotted with GraphPad Prism 8 (RRID:SCR_002798). For the primary screen, data from up to six independent biological replicates were combined (all normalized to the wildtype LRRK2 values for each replicate). For the secondary screen, data from two independent biological replicates (each performed in duplicate) were combined (all normalized to the wildtype LRRK2 values for each replicate). For the immunoblotting data obtained from the primary screen, outliers of LRRK2 variant activity were determined using an arbitrary cut-off of LRRK2 expression 1.9-fold higher or lower than wildtype LRRK2 expression (designated as 1) and are presented in Figures 1C and 2 as open circle data points and excluded from the variant mean. LRRK2 variants that were expressed less than ∼1.9 relative to wildtype LRRK2, or greater than ∼0.53 relative to wildtype LRRK2, were considered true representations of variant activity and are presented in Figures 1C and 2 as closed circles.

#### Statistical analysis

Gathered data either from immunoblotting or cell counting was analyzed using GraphPad Prism 8 (RRID:SCR_002798). One- or multi-way ANOVA with Dunnett's multiple comparisons *post hoc* test was used to determine statistical significance and approximate *P* values for each value compared with the control mean — wildtype LRRK2.

## Data Availability

All the primary data that is presented in this study has been deposited on the Zenodo data repository (10.5281/zenodo.6401193). All plasmids and antibodies (and associated datasheets) generated at the MRC Protein Phosphorylation and Ubiquitylation Unit at the University of Dundee can be requested through our website https://mrcppureagents.dundee.ac.uk/.

## Open Access

Open access for this article was enabled by the participation of the Univesity of Dundee in an all-inclusive *Read & Publish* agreement with Portland Press and the Biochemical Society under a transformative agreement with JISC.

## Abbreviations

AFDB: AlphaFold database
ANK: ankyrin
ARM: armadillo
CD: Crohn's disease
HH: hinge-helix
LRR: leucine-rich repeats
PBS: phosphate buffered saline
PD: Parkinson's disease
PDB: Protein Data Bank
PEI: polyethylenimine
