# Supplementary material for: Impact of 100 LRRK2 variants linked to Parkinson's disease on kinase activity and microtubule binding
Source: Biochem J. 2022 Sep 6;479(17):1759–83. doi: 10.1042/BCJ20220161 (PMC9472821; doi:10.1042/BCJ20220161)
Supplement: Supplementary Material [file BCJ-479-1759-s1.pdf]

Supplementary Section

Impact of 100 LRRK2 variants linked to Parkinson's Disease on kinase activity and microtubule binding

Alexia F Kalogeropulou[1,2*], Elena Purlyte[1,6*], Francesca Tonelli[1,2*], Sven M Lange [1], Melanie Wightman [1], Alan R Prescott [3], Shalini Padmanabhan[4,] Esther Sammler [1,2,5], Dario R Alessi[1,2#]

1. MRC Protein Phosphorylation and Ubiquitylation Unit, School of Life Sciences, University of Dundee, Dow Street, Dundee, United Kingdom.

2. Aligning Science Across Parkinson's (ASAP) Collaborative Research Network, Chevy Chase, MD, 20815, USA.

3. Dundee Imaging Facility, School of Life Sciences, University of Dundee, Dundee DD1 5EH, United Kingdom.

4. The Michael J. Fox Foundation for Parkinson's Research, NY, USA.

5. Molecular and Clinical Medicine, Ninewells Hospital and Medical School, University of Dundee, Dundee, DD1 9SY, UK.

6. Current address: Department of Molecular Biology, University of Texas Southwestern Medical Center, Dallas, TX 75390, USA.

Supplementary Figure 1. LRRK2 S1647T does not impact wildtype or pathogenic R1441G or G2019S LRRK2 activity. FLAG empty vector, FLAG-tagged LRRK2 wildtype, and the indicated variants were expressed in HEK293 cells. Cells were lysed 24 hours post-transfection and were analysed by quantitative immunoblotting with the indicated antibodies. Each lane represents a different dish of cells. The ratios of phospho-Rab10 Thr73/total Rab10 and phospho-LRRK2 Ser935/total LRRK2 were normalized to wildtype LRRK2 values. Quantified data are presented as mean ± SD and are representative of two independent experiments.

Supplementary Figure 2. Primary quantitative immunoblot screen to assess activity of selected PD and CD-associated LRRK2 variants. (A) FLAG-tagged LRRK2 wildtype, kinase dead (KD = D2017A) and the indicated LRRK2 variants were transiently expressed in HEK293 cells. Cells were lysed 24 hours post-transfection and analysed by quantitative immunoblotting using the indicated antibodies. Immunoblot figure is representative of 6 independent biological replicates. Quantification of the combined immunoblotting data from all replicates is presented in Fig 1C, Figure 2A, Figure 2B and Figure 2C. (B) FLAG-tagged LRRK2 wildtype, kinase dead (KD = D2017A) and the indicated variants were transiently expressed in HEK293 cells. Each lane represents a different dish of cells. Cell lysate was analysed by quantitative immunoblotting with the indicated antibodies. The ratios of phospho-Rab10 Thr73/total Rab10, phospho-LRRK2 Ser935-955-973/total LRRK2,

phospho-LRRK2 Ser1292/total LRRK2, were normalized to the average of wildtype LRRK2 values. Quantified data are presented as mean ± SD.

Supplementary Figure 3. Quantitative analysis of biomarker phosphorylation of selected PD and CD-associated LRRK2 variants assessed in primary immunoblot screens. FLAG-tagged LRRK2 wildtype, kinase dead (KD = D2017A) and the indicated LRRK2 variants were transiently expressed in HEK293 cells. Cells were lysed 24 hours post-transfection and analysed by quantitative immunoblotting. Quantified immunoblotting data are presented as the ratio of LRRK2 pSer955/total LRRK2 (A) and LRRK2 pSer973/total LRRK2 (B), normalized to the average of LRRK2 wildtype values for each replicate (mean ± SD). Dashed lines segment the graphs into the corresponding regions of LRRK2 as listed in the domain schematic above panel.

Supplementary Figure 4. Rab10 phosphorylation mediated by selected PD and CD LRRK2 variants is reduced with MLi-2 LRRK2 inhibitor. FLAG-tagged LRRK2 wildtype, kinase dead (KD = D2017A) and the indicated LRRK2 variants were transiently expressed in HEK293 cells for 24 hours. Three hours prior to cell lysis, cells were treated with vehicle (0.1% v/v DMSO) or 100 nM MLi-2. Cell lysates were subjected to quantitative immunoblotting with the indicated antibodies.

Supplementary Figure 5. The protective LRRK2 R1398H variant does not impact wildtype or pathogenic LRRK2 R1441G, Y1699C, or G2019S activity. FLAG-tagged LRRK2 wildtype, kinase dead (KD = D2017A) and the indicated LRRK2 variants were transiently expressed in HEK293 cells for 24 hours. Each lane represents a different dish of cells. Cell lysates were subjected to quantitative immunoblotting with the indicated antibodies. Quantified immunoblotting data are presented as the ratios of phospho-Rab10 Thr73/total Rab10 and phospho-LRRK2 Ser935/total LRRK2, normalized to the average of wildtype LRRK2 values (mean ± SD). Quantified data are representative of two independent experiments.

Supplementary Figure 6. Analysis of *in vitro* LRRK2 kinase activity against recombinant Rab8A of selected LRRK2 variants. FLAG-tagged LRRK2 wildtype, kinase dead (KD = D2017A) and the indicated LRRK2 variants were transiently expressed in HEK293 cells for 24 hours. (A) Whole cell lysates were analysed by quantitative immunoblotting using the indicated antibodies. (B) FLAG-LRRK2 was immunoprecipitated from whole cell lysates and subjected to an *in vitro* kinase reaction in the presence of recombinant Rab8A. Kinase reaction products were analysed by quantitative immunoblotting using the indicated antibodies.

Supplementary Figure 7. COR_B and kinase domain LRRK2 variants reproducibly enhance *in vitro* LRRK2 kinase activity. FLAG-tagged LRRK2 wildtype, kinase dead (KD = D2017A) and the indicated LRRK2 variants were transiently expressed in HEK293 cells for 24 hours. (A) Whole cell lysates were analysed by quantitative immunoblotting using the indicated antibodies. (B) FLAG-LRRK2 was immunoprecipitated from whole cell lysates and subjected to an *in vitro* kinase reaction in the presence of recombinant Rab8A. Kinase reaction products were analysed by quantitative immunoblotting using the indicated antibodies. Quantified immunoblotting data are presented as ratios of phospho-LRRK2 Thr1357/total LRRK2, phospho-LRRK2 Thr1503/total LRRK2, or phospho-Rab8A Thr72/total Rab8A, normalized to the average of wildtype LRRK2 values (mean ± SD).

Supplementary Figure 8. Combination of COR_B and kinase domain LRRK2 mutations further enhances *in vitro* LRRK2 kinase activity. FLAG-tagged LRRK2 wildtype, kinase dead (KD = D2017A) and the indicated LRRK2 variants were transiently expressed in HEK293 cells for 24 hours. (A) Whole cell lysates were analysed by quantitative immunoblotting using the indicated antibodies. Quantified immunoblotting data are presented as ratios of phospho-LRRK2 Ser935/total LRRK2, phospho-Rab10 Thr73/total Rab10, and total LRRK2/Tubulin. (B) FLAG-LRRK2 was immunoprecipitated from whole cell lysates and subjected to an *in vitro* kinase reaction in the presence of recombinant Rab8A. Kinase reaction products were analysed by quantitative immunoblotting using the indicated antibodies. Quantified data are presented in Fig 4E.

Supplementary Figure 9. Activation of selected LRRK2 variants by Rab29. FLAG-tagged LRRK2 wildtype, kinase dead (KD = D2017A) and the indicated variants were transiently expressed in HEK293 cells with HA empty vector or HA-tagged Rab29. 24 hours post-transfection, cells were lysed and analysed by quantitative immunoblotting with the indicated antibodies. Quantified data are presented in Figure 5.

Supplementary Figure 10. Superposition of full-length LRRK2 electron density map (PDB 7LHW) on Alphafold LRRK2 model. Alphafold model of LRRK2 ARM domain has a high local confidence score (pLDDT) and agrees well with experimental data. The ARM domain of the Alphafold model of LRRK2 (residues 159-511, AFDB AF-Q5S007-F1-model_v1) was coloured by pLDDT and fitted into the experimental cryo-EM map of full-length LRRK2 (grey, EMD-23352) using the UCSF ChimeraX "Fit in Map" tool. The remaining C-terminal LRRK2 residues [540-2527] are shown in green (PDB 7LHW).

Supplementary Figure 1

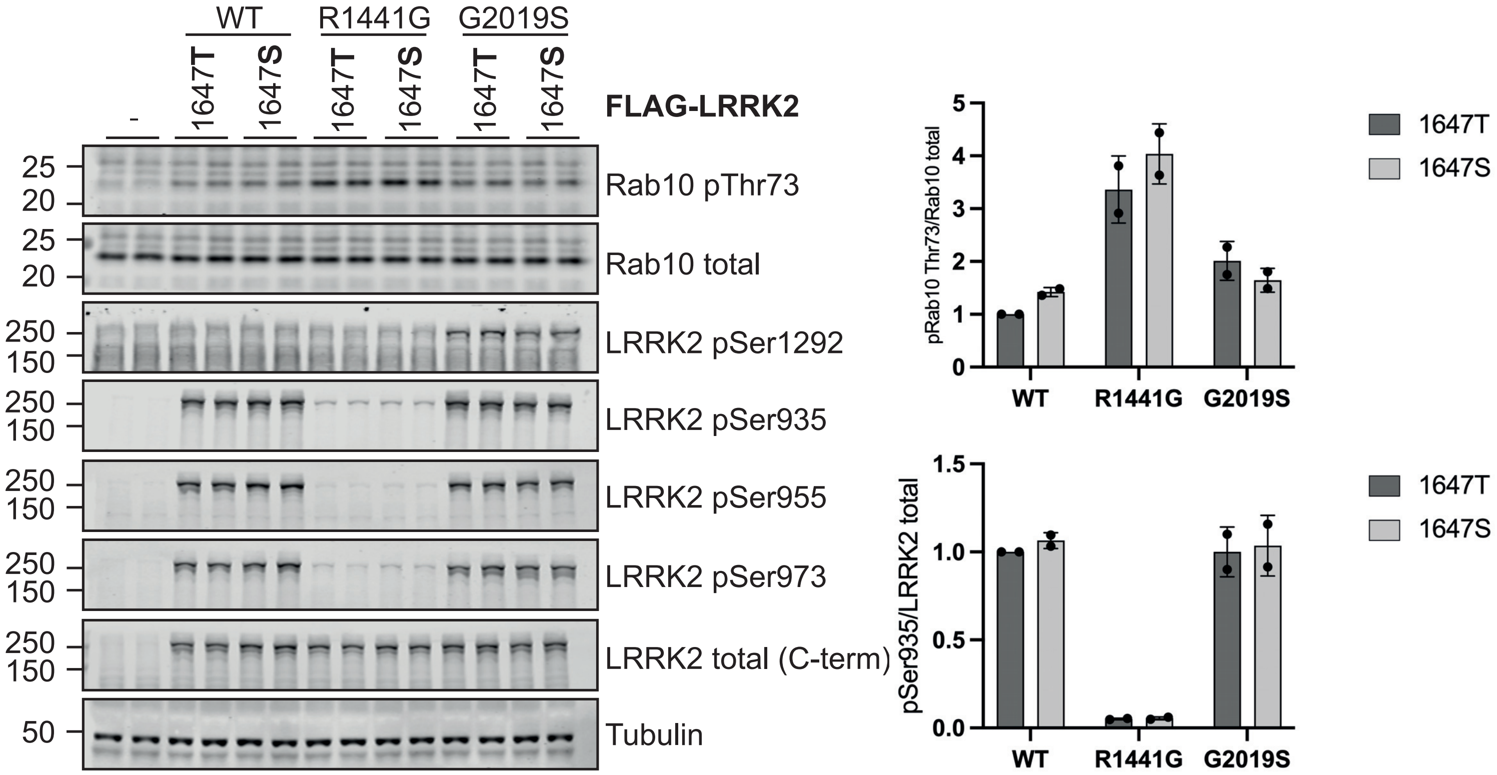

Supplementary Figure 2

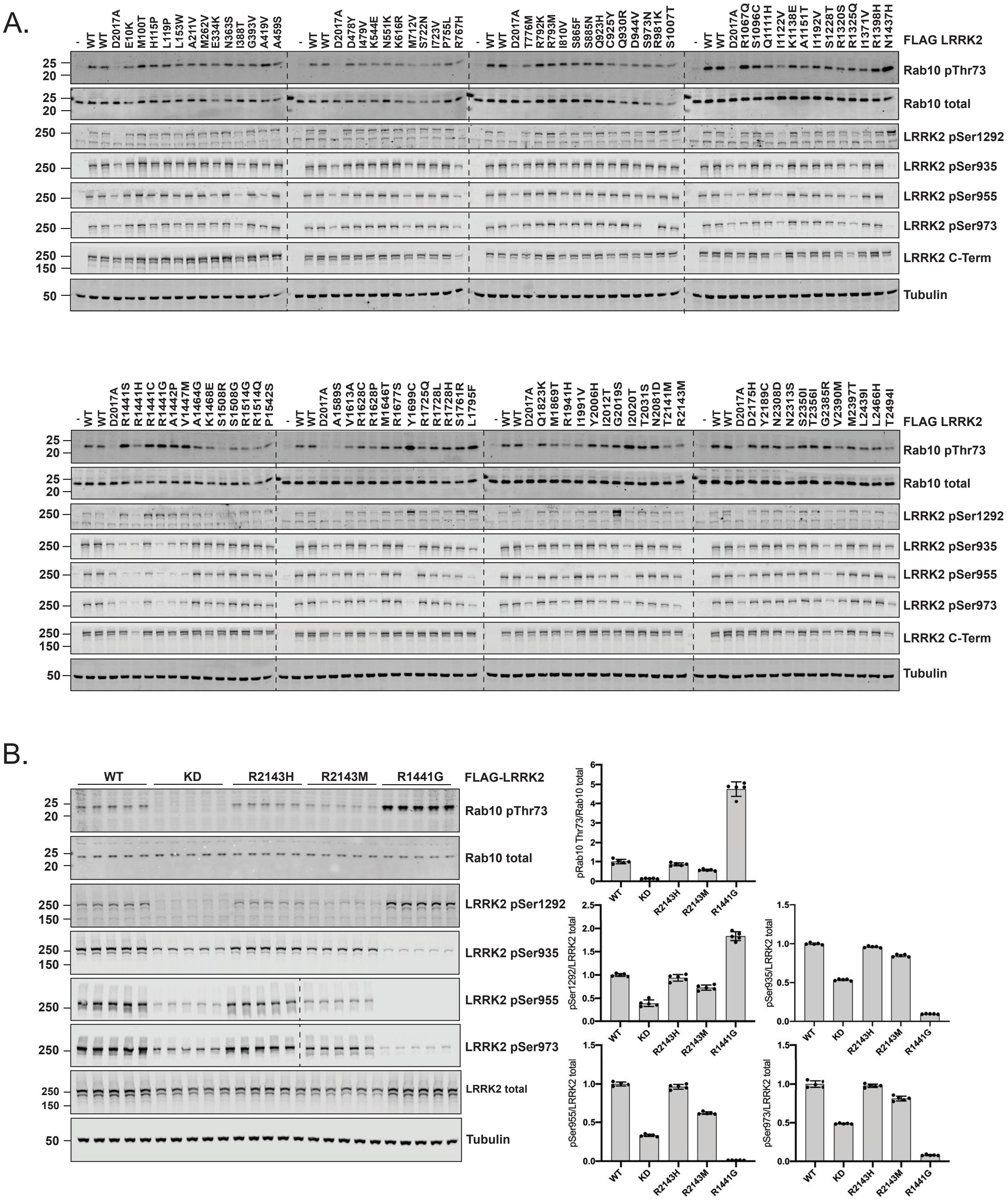

Supplementary Figure 3

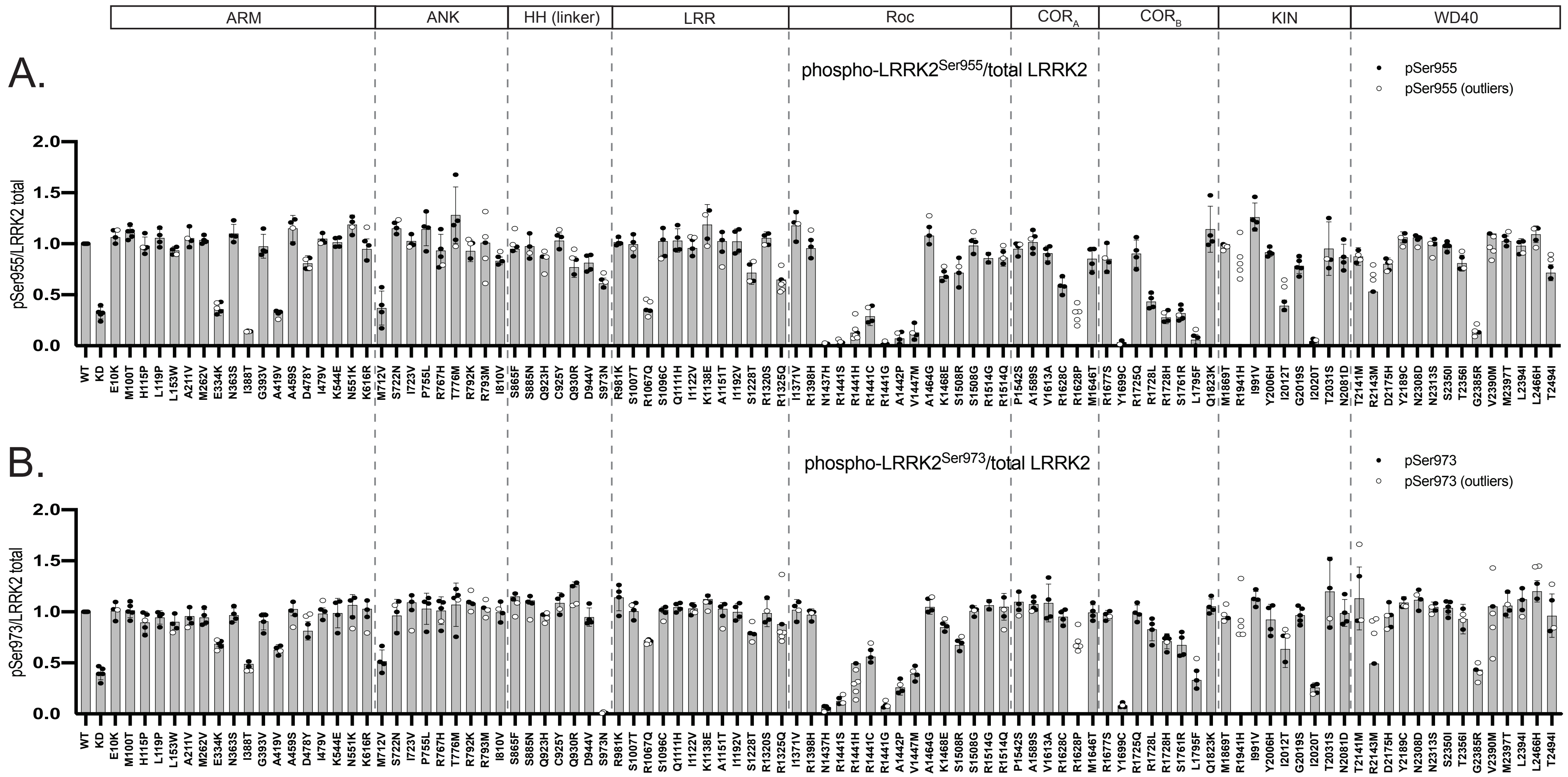

Supplementary Figure 4

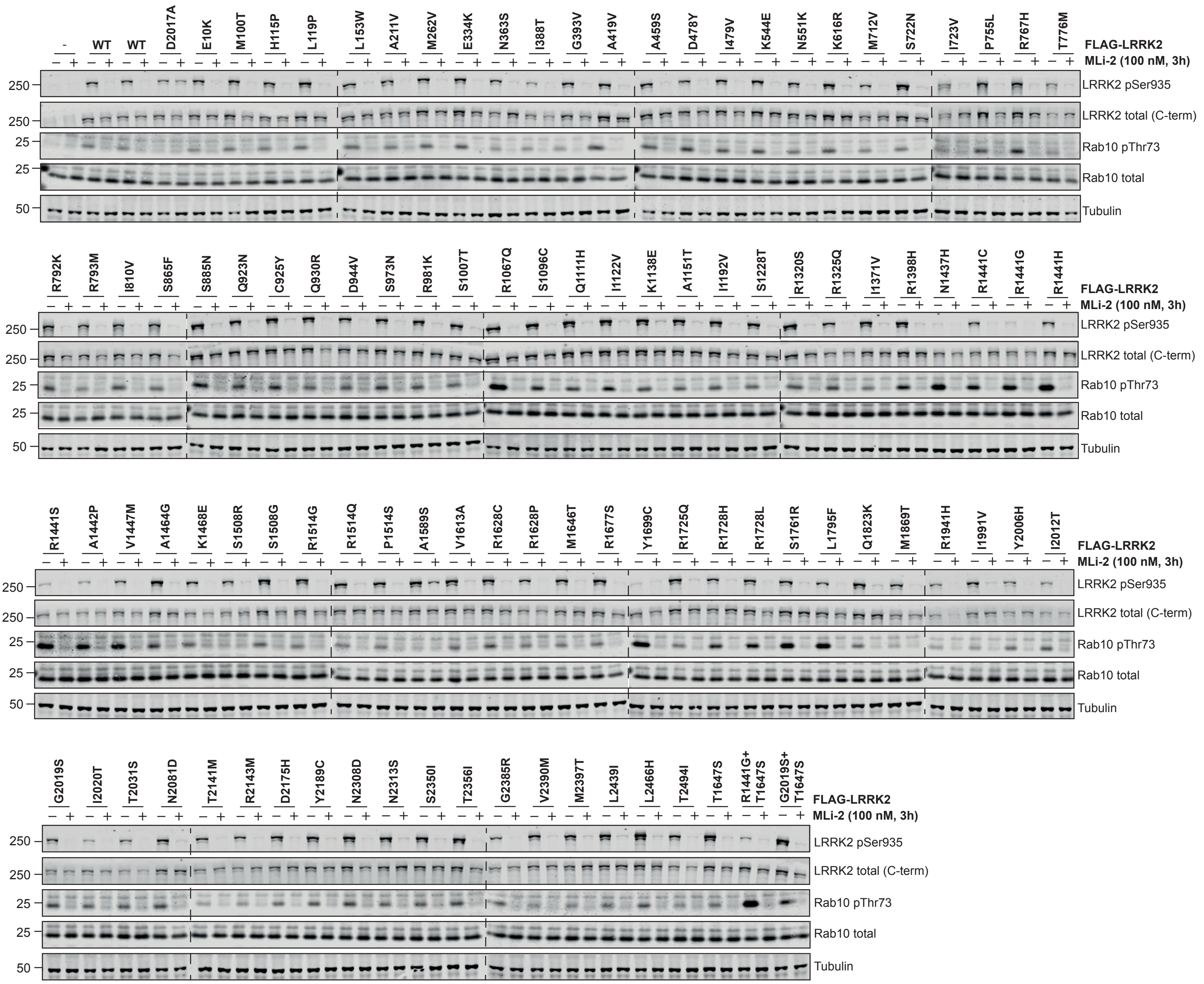

Supplementary Figure 5

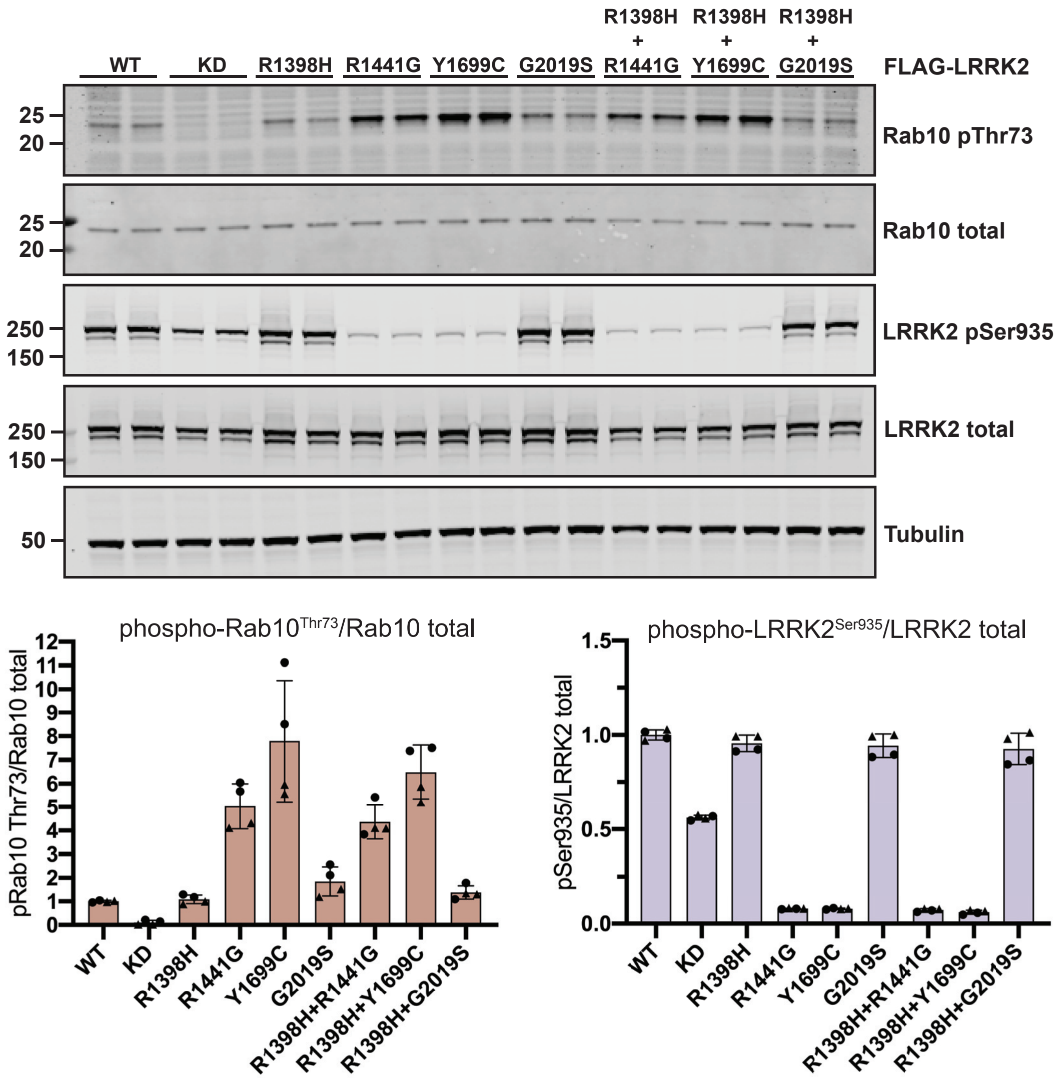

Supplementary Figure 6

A.

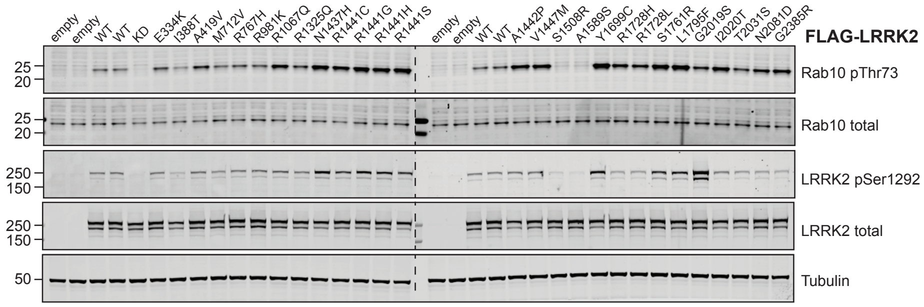

B.

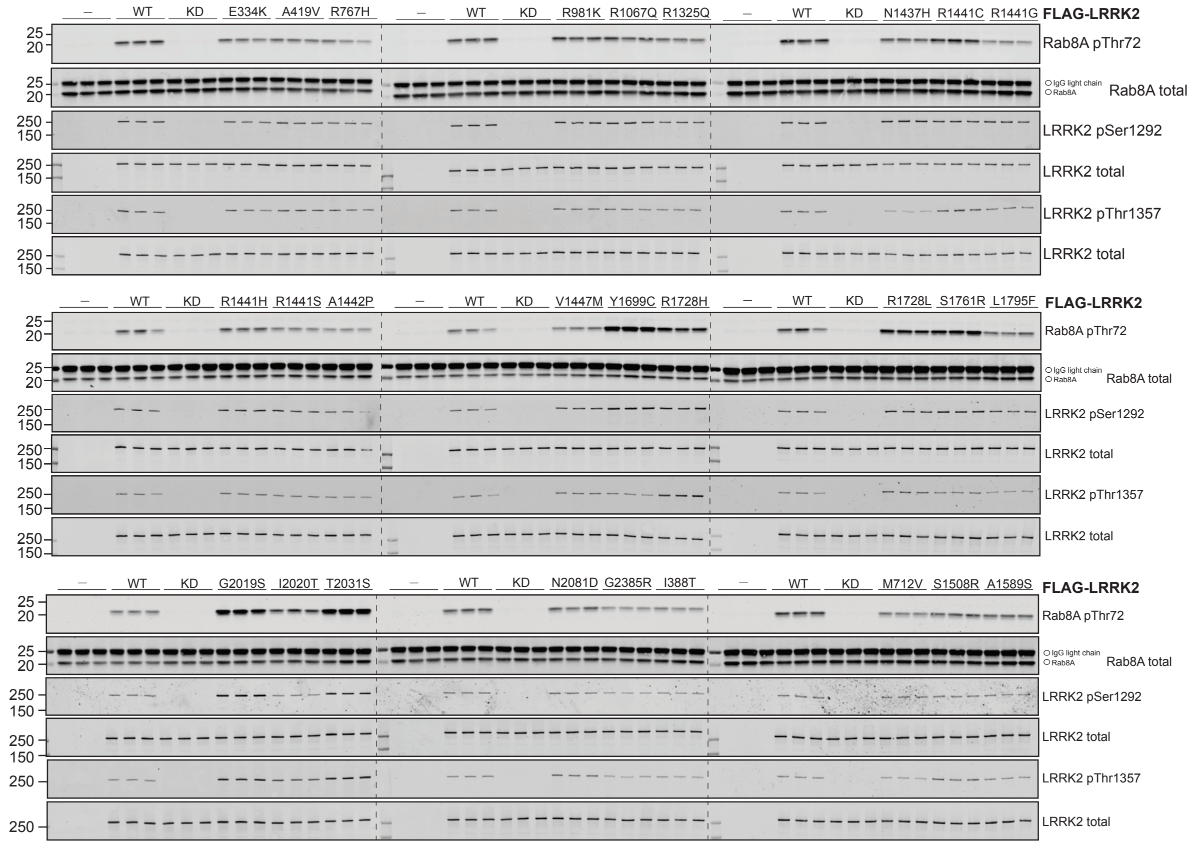

Supplementary Figure 7

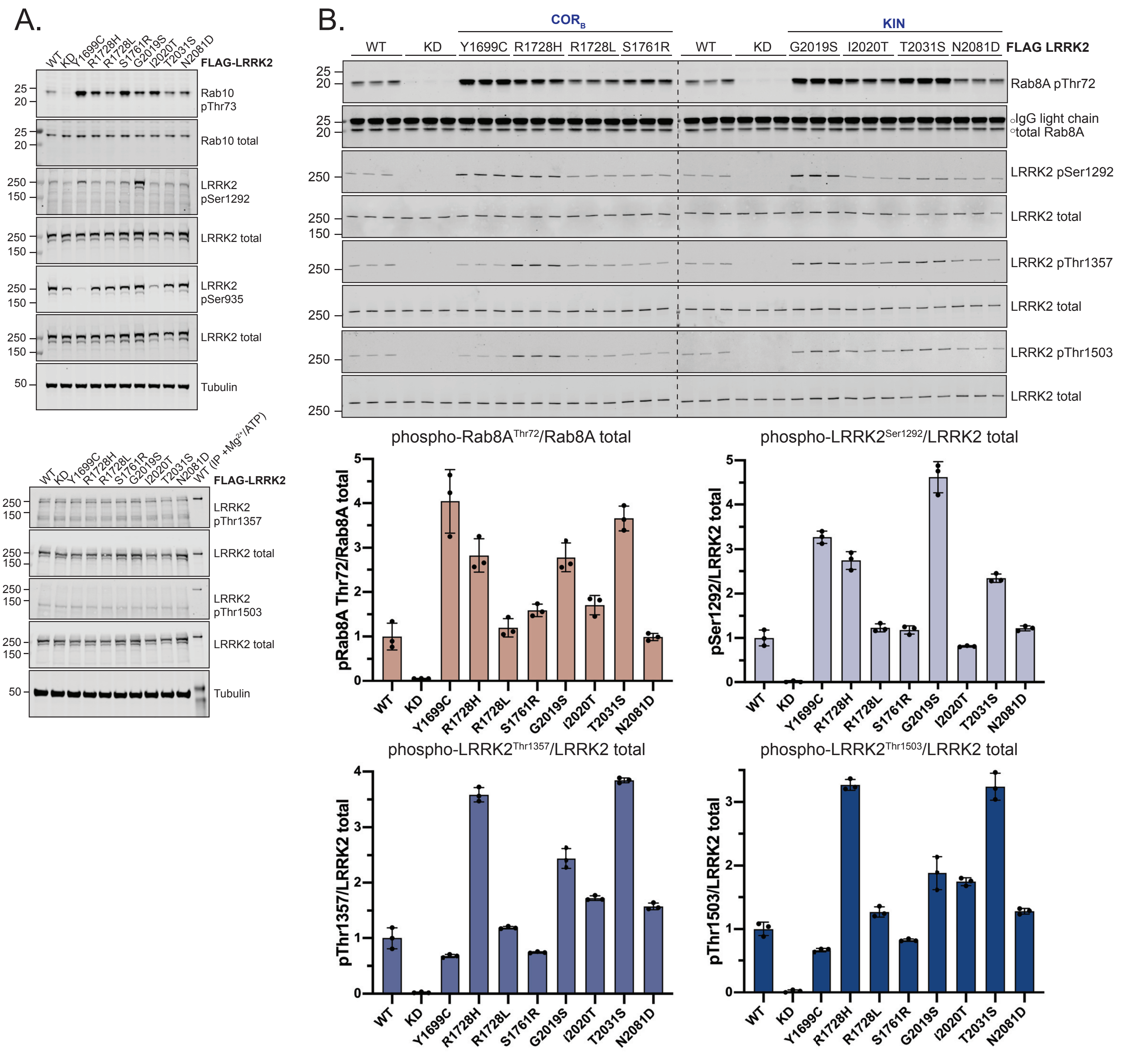

Supplementary Figure 8

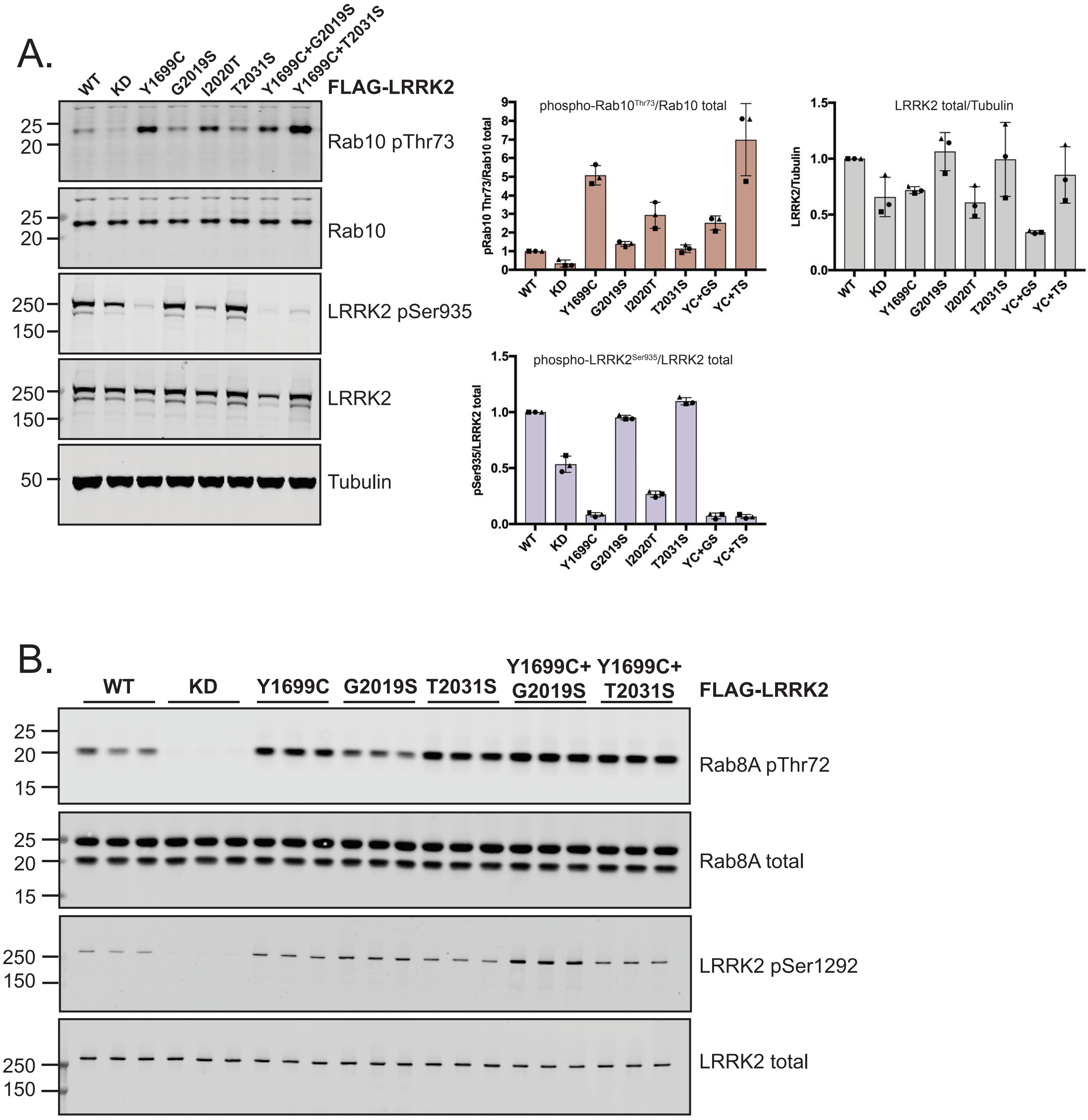

Supplementary Figure 9

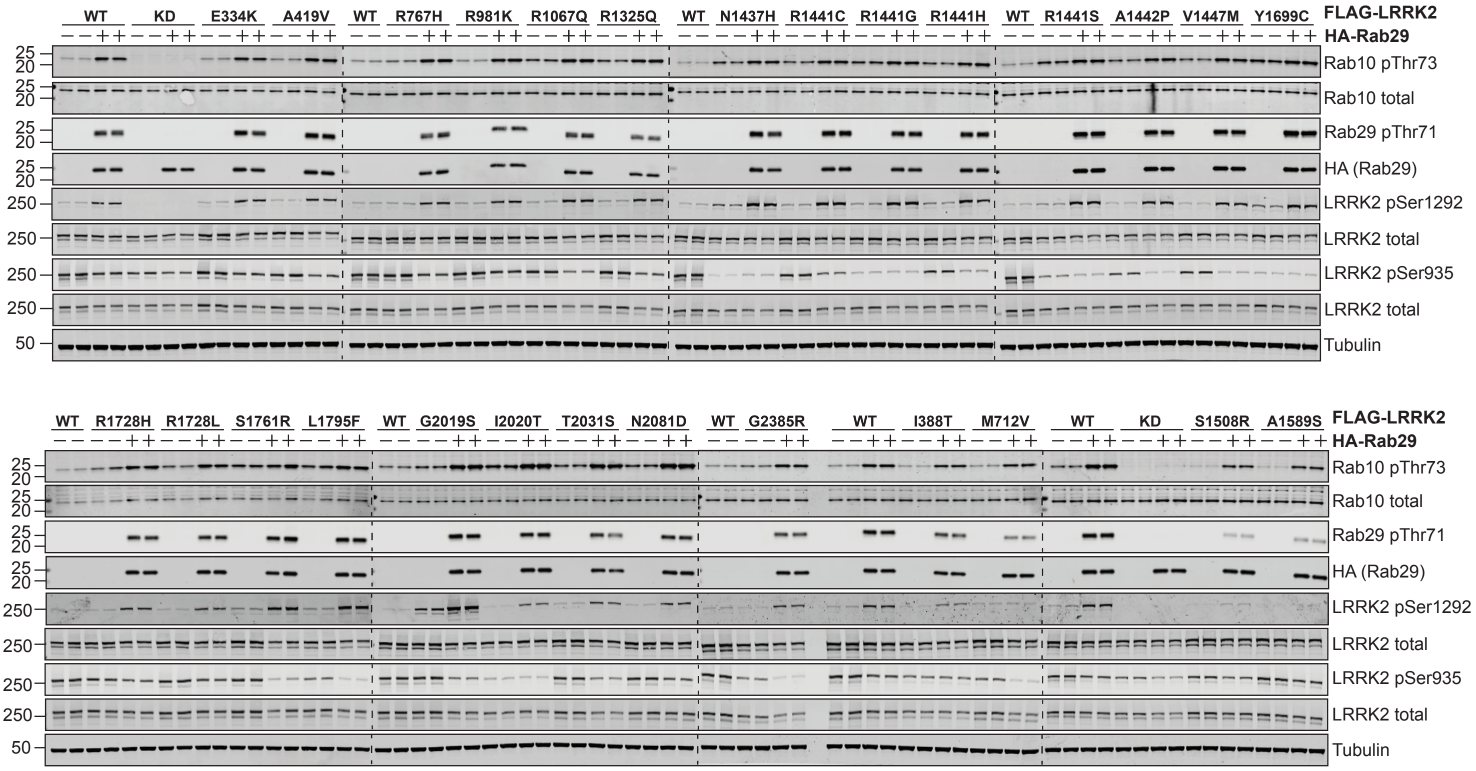

Supplementary Figure 10

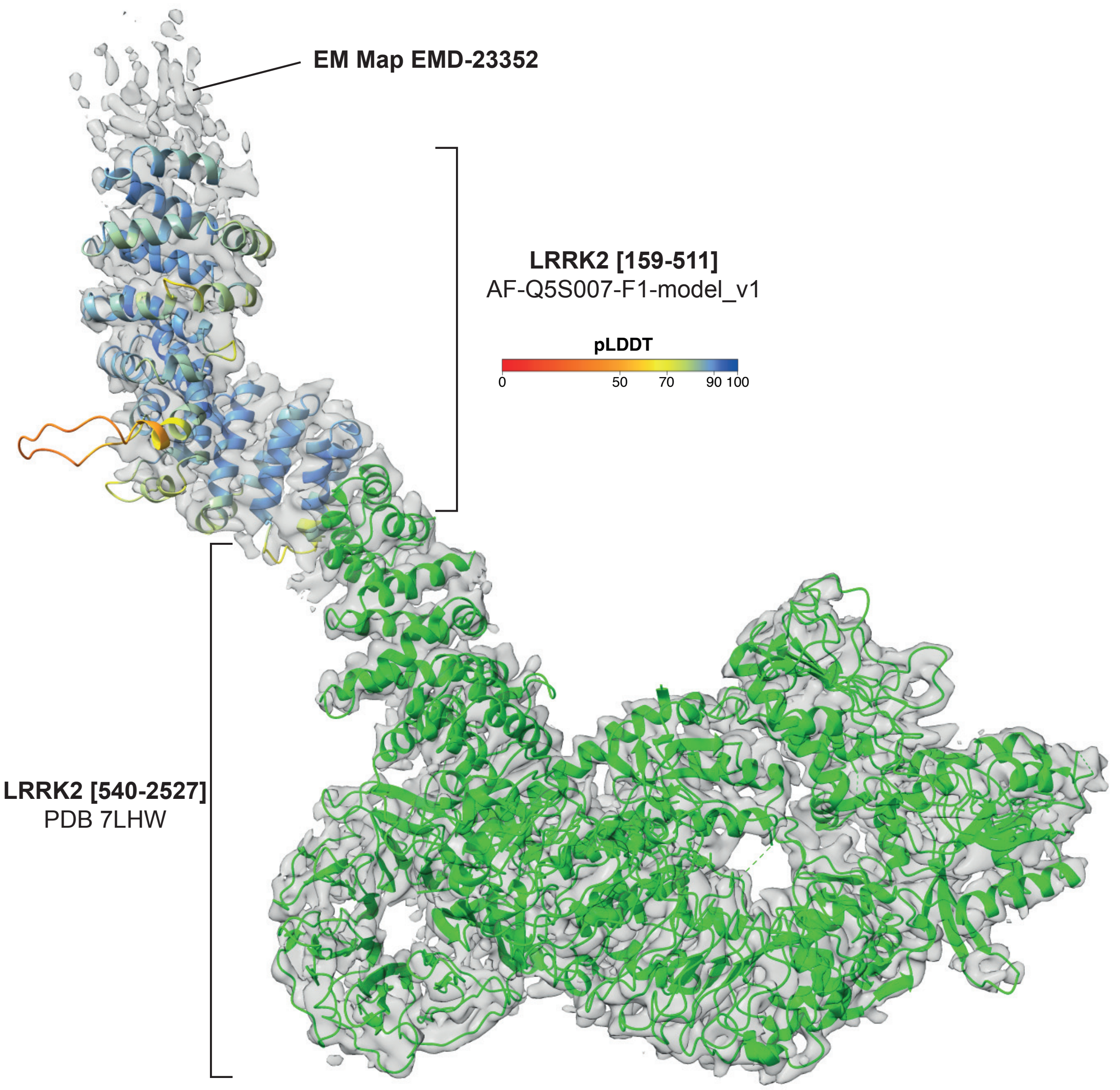

Supplementary Table 1

No.	*Mutation (Single Letter)*	*Mutation*	*Domain location*	*REVEL score*	*Conservation Score*	*Reference*
1	E10K	p.Glu10Lys	ARM	0.207	1	[1]
2	M100T	p.Met100Thr	ARM	0.209	2	[2]
3	H115P	p.His115Pro	ARM	0.566	9	Unpublished*
4	L119P	p.Leu119Pro	ARM	0.464	8	[3]*
5	L153W	p.Leu153Trp	ARM	0.277	8	[2]
6	A211V	p.Ala211Val	ARM	0.206	6	[4]
7	M262V	p.Met262Val	ARM	0.013	1	Unpublished*
8	E334K	p.Glu334Lys	ARM	0.194	6	[1]
9	N363S	p.Asn363Ser	ARM	0.039	5	[5]
10	I388T	p.Ile388Thr	ARM	0.161	7	Unpublished*
11	G393V	p.Gly393Val	ARM	0.124	1	Unpublished*
12	A419V	p.Ala419Val	ARM	0.175	7	[6]
13	A459S	p.Ala459Ser	ARM	0.089	6	[2]
14	D478Y	p.Asp478Tyr	ARM	0.369	9	Unpublished*
15	I479V	p.Ile479Val	ARM	0.026	1	Unpublished*
16	K544E	p.Lys544Glu	ARM	0.288	2	[4]
17	N551K	p.Asn551Lys	ARM	0.248	9	[6]*
18	K616R	p.Lys616Arg	ARM	0.264	6	[7]
19	M712V	p.Met712Val	ANK	0.228	6	[8]
20	S722N	p.Ser722Asn	ANK	0.098	5	[2]
21	I723V	p.Ile723Val	ANK	0.045	1	[3]*
22	P755L	p.Pro755Leu	ANK	0.173	5	[6]
23	R767H	p.Arg767His	ANK	0.157	7	[9]*
24	T776M	p.Thr776Met	ANK	0.046	1	Unpublished*
25	R792K	p.Arg792Lys	ANK	0.023	1	[2]
26	R793M	p.Arg793Met	ANK	0.305	8	[10]
27	I810V	p.Ile810Val	LRR	0.016	2	[5]
28	S865F	p.Ser865Phe	LRR	0.149	1	[11]*
29	S885N	p.Ser885Asn	LRR	0.025	2	[9]
30	Q923H	p.Gln923His	LRR	0.272	1	[12]*
31	C925Y	p.Cys925Tyr	LRR	0.039	1	[2]
32	Q930R	p.Gln930Arg	LRR	0.286	1	[10]
33	D944V	p.Asp944Val	LRR	0.14	1	Unpublished*
34	S973N	p.Ser973Asn	LRR	0.032	6	[13]
35	R981K	p.Arg981Lys	LRR	0.062	3	[2]
36	S1007T	p.Ser1007Thr	LRR	0.054	2	[2]
37	R1067Q	p.Arg1067Gln	LRR	0.282	8	[14]
38	S1096C	p.Ser1096Cys	LRR	0.204	2	[10]
39	Q1111H	p.Gln1111His	LRR	0.219	8	[1]
40	I1122V	p.Ile1122Val	LRR	0.244	3	[15]

Supplementary Table 1

41	K1138E	p.Lys1138Glu	LRR	0.354	7	Unpublished from Tony Segal
42	A1151T	p.Ala1151Thr	LRR	0.029	1	[16]
43	I1192V	p.Ile1192Val	LRR	0.215	5	[1]
44	S1228T	p.Ser1228Thr	LRR	0.307	7	[10]*
45	R1320S	p.Arg1320Ser	LRR	0.319	9	[3]
46	R1325Q	p.Arg1325Gln	LRR	0.553	9	[5,17]
47	I1371V	p.Ile1371Val	ROC	0.453	8	[18]
48	R1398H	p.Arg1398His	ROC	0.369	7	[6]*
49	N1437H	p.Asn1437His	ROC	0.603	9	[19]
50	R1441S	p.Arg1441Ser	ROC	0.660	7	[20]
51	R1441H	p.Arg1441His	ROC	0.635	7	[21]
52	R1441C	p.Arg1441Cys	ROC	0.727	7	[15]
53	R1441G	p.Arg1441Gly	ROC	0.705	7	[18]
54	A1442P	p.Ala1442Pro	ROC	0.783	9	[22]
55	V1447M	p.Val1447Met	ROC	0.749	9	[2]
56	A1464G	p.Ala1464Gly	ROC	0.209	3	[23]
57	K1468E	p.Lys1468Glu	ROC	0.585	9	[17]*
58	S1508R	p.Ser1508Arg	ROC	0.522	6	Unpublished- online PhD presentation (2009)
59	S1508G	p.Ser1508Gly	ROC	0.390	6	[24]
60	R1514G	p.Arg1514Gly	COR_A	0.354	9	Unpublished
61	R1514Q	p.Arg1514Gln	COR_A	0.1	9	[1,21,25]*
62	P1542S	p.Pro1542Ser	COR_A	0.228	4	[21]*
63	A1589S	p.Ala1589Ser	COR_A	0.253	8	Unpublished*
64	V1613A	p.Val1613Ala	COR_A	0.585	5	[26]
65	R1628C	p.Arg1628Cys	COR_A	0.451	8	[27]
66	R1628P	p.Arg1628Pro	COR_A	0.546	8	[21]*
67	M1646T	p.Met1646Thr	COR_A	0.184	3	[21]*
68	S1647T	p.Ser1647Thr	COR_A	0.086	2	[28]**
69	R1677S	p.Arg1677Ser	COR_B	0.386	7	[2]
70	Y1699C	p.Tyr1699Cys	COR_B	0.87	9	[15]
71	R1725Q	p.Arg1725Gln	COR_B	0.095	6	[29]
72	R1728L	p.Arg1728Leu	COR_B	0.763	8	[8]
73	R1728H	p.Arg1728His	COR_B	0.721	8	[8]
74	S1761R	p.Ser1761Arg	COR_B	0.521	8	[30]
75	L1795F	p.Leu1795Phe	COR_B	0.638	9	[1]
76	Q1823K	p.Gln1823Lys	COR_B	0.188	2	[29]
77	M1869T	p.Met1869Thr	COR_B	0.514	6	[21]
78	R1941H	p.Arg1941His	KIN	0.24	4	[31]*
79	I1991V	p.Ile1991Val	KIN	0.448	8	[24]
80	Y2006H	p.Tyr2006His	KIN	0.301	8	[32]
81	I2012T	p.Ile2012Thr	KIN	0.664	8	[33]
82	G2019S	p.Gly2019Ser	KIN	0.97	9	[34–36]*

Supplementary Table 1

83	I2020T	p.Ile2020Thr	KIN	0.957	9	[15,37]
84	T2031S	p.Thr2031Ser	KIN	0.280	9	[32]
85	N2081D	p.Asn2081Asp	KIN	0.138	9	[3]*
86	T2141M	p.Thr2141Met	KIN	0.342	5	[8]
87	R2143H	p.Arg2143His	WD40	0.277	1	[8]
88	R2143M	p.Arg2143Met	WD40	N/A	1	[38]***
89	D2175H	p.Asp2175His	WD40	0.307	1	[29]
90	Y2189C	p.Tyr2189Cys	WD40	0.394	1	[17]
91	N2308D	p.Asn2308Asp	WD40	0.025	1	[2]
92	N2313S	p.Asn2313Ser	WD40	0.043	3	[2]
93	S2350I	p.Ser2350Ile	WD40	0.121	5	[2]
94	T2356I	p.Thr2356Ile	WD40	0.154	6	[31]
95	G2385R	p.Gly2385Arg	WD40	0.044	5	[21,39] *
96	V2390M	p.Val2390Met	WD40	0.205	4	[40]
97	M2397T	p.Met2397Thr	WD40	0.098	1	[6]*
98	L2439I	p.Leu2439Ile	WD40	0.135	7	[29]
99	L2466H	p.Leu2466His	WD40	0.311	2	[8]
100	T2494I	p.Thr2494Ile	WD40	0.065	5	[41]

*LRRK2 variants disclosed by Michael J Fox Foundation through Parkinson’s Progression Markers Initiative (PPMI) clinical database.

**S1647T is a common variant that has been reported by several studies to be associated with PD risk. All LRRK2 cDNA constructs in this study contain the S1647T variant. The activity of S1647T was analysed in parallel with T1647S.

***R2143M was analysed in this study based on work undertaken previously in our laboratory [42], but no primary reference could be identified for this variant.

Supplementary Table 1

References

1 Nichols, W. C., Elsaesser, V. E., Pankratz, N., Pauciulo, M. W., Marek, D. K., Halter, C. A., Rudolph, A., Shults, C. W. and Foroud, T. (2007) LRRK2 mutation analysis in Parkinson disease families with evidence of linkage to PARK8. Neurology **69**, 1737–1744.

2 Zhang, J. R., Jin, H., Li, K., Mao, C. J., Yang, Y. P., Wang, F., Gu, C. C., Zhang, H. J., Chen, J. and Liu, C. F. (2018) Genetic analysis of LRRK2 in Parkinson's disease in Han Chinese population. Neurobiol. Aging, Elsevier Inc **72**, 187.e5-187.e10.

3 Ross, O. A., Soto-Ortolaza, A. I., Heckman, M. G., Aasly, J. O., Abahuni, N., Annesi, G., Bacon, J. A., Bardien, S., Bozi, M., Brice, A., et al. (2011) Association of LRRK2 exonic variants with susceptibility to Parkinson's disease: A case-control study. Lancet Neurol. **10**, 898–908.

4 Xiromerisiou, G., Hadjigeorgiou, G. M., Gourbali, V., Johnson, J., Papakonstantinou, I., Papadimitriou, A. and Singleton, A. B. (2007) Screening for SNCA and LRRK2 mutations in Greek sporadic and autosomal dominant Parkinson's disease: Identification of two novel LRRK2 variants. Eur. J. Neurol. **14**, 7–11.

5 Lesage, S., Condroyer, C., Lannuzel, A., Lohmann, E., Troiano, A., Tison, F., Damier, P., Thobois, S., Ouvrard-Hernandez, A. M., Rivaud-Péchoux, S., et al. (2009) Molecular analyses of the LRRK2 gene in European and North African autosomal dominant Parkinson's disease. J. Med. Genet. **46**, 458–464.

6 Di Fonzo, A., Tassorelli, C., De Mari, M., Chien, H. F., Ferreira, J., Rohé, C. F., Riboldazzi, G., Antonini, A., Albani, G., Mauro, A., et al. (2006) Comprehensive analysis of the LRRK2 gene in sixty families with Parkinson's disease. Eur. J. Hum. Genet. **14**, 322–331.

7 Wang, L., Guo, J. feng, Nie, L. luo, Xu, Q., Zuo, X., Sun, Q. ying, Yan, X. xiang and Tang, B. sha. (2010) A novel LRRK2 mutation in a mainland Chinese patient with familial Parkinson's disease. Neurosci. Lett. **468**, 198–201.

8 Paisán-Ruíz, C., Nath, P., Washecka, N., Gibbs, J. R. and Singleton, A. B. (2008) Comprehensive analysis of LRRK2 in publicly available Parkinson's disease cases and neurologically normal controls. Hum. Mutat. **29**, 485–490.

9 Wu, Y. R., Chang, K. H., Chang, W. T., Hsiao, Y. C., Hsu, H. C., Jiang, P. R., Chen, Y. C., Chao, C. Y., Chang, Y. C., Lee, B. H., et al. (2013) Genetic variants of LRRK2 in Taiwanese Parkinson's disease. PLoS One **8**, 11–14.

10 Berg, D., Schweitzer, K., Leitner, P., Zimprich, A., Lichtner, P., Belcredi, P., Brussel, T., Schulte, C., Maass, S. and Nagele, T. (2005) Type and frequency of mutations in the LRRK2 gene in familial and sporadic Parkinson's disease. Brain **128**, 3000–3011.

11 Al-Mubarak, B. R., Bohlega, S. A., Alkhairallah, T. S., Magrashi, A. I., AlTurki, M. I., Khalil, D. S., AlAbdulaziz, B. S., Al-Shaar, H. A., Mustafa, A. E., Alyemni, E. A., et al. (2015) Parkinson's disease in Saudi patients: A genetic study. PLoS One **10**, 1–14.

12 Camargos, S. T., Dornas, L. O., Momeni, P., Lees, A., Hardy, J., Singleton, A. and Cardoso, F. (2010) Familial Parkinsonism and early onset Parkinson's disease in a Brazilian movement disorders clinic: Phenotypic characterization and frequency of SNCA, PRKN, PINK1, and LRRK2 mutations. Mov. Disord. **24**, 662–666.

13 Haubenberger, D., Bonelli, S., Hotzy, C., Leitner, P., Lichtner, P., Samal, D., Katzenschlager, R., Djamshidian, A., Brücke, T., Steffelbauer, M., et al. (2007) A novel LRRK2 mutation in an Austrian cohort of patients with Parkinson's disease. Mov. Disord. **22**, 1640–1643.

14 Skipper, L., Shen, H., Chua, E., Bonnard, C., Kolatkar, P., Tan, L. C. S., Jamora, R. D., Puvan, K., Puong, K. Y., Zhao, Y., et al. (2005) Analysis of LRRK2 functional domains in nondominant Parkinson disease. Neurology **65**, 1319–1321.

Supplementary Table 1

15 Zimprich, A., Biskup, S., Leitner, P., Lichtner, P., Farrer, M., Lincoln, S., Kachergus, J., Hulihan, M., Uitti, R. J., Calne, D. B., et al. (2004) Mutations in LRRK2 cause autosomal-dominant parkinsonism with pleomorphic pathology. Neuron **44**, 601–607.

16 Schlitter, A., Woitalla, D., Mueller, T., Epplen, J. and Dekomien, G. (2006) The LRRK2 gene in Parkinson's disease: mutation screening in patients from Germany. J. Neurol. Neurosurg. Psychiatry **77**, 891–892.

17 Nuytemans, K., Rademakers, R., Theuns, J., Pals, P., Engelborghs, S., Pickut, B., de Pooter, T., Peeters, K., Mattheijssens, M., Van den Broeck, M., et al. (2008) Founder mutation p.R1441C in the leucine-rich repeat kinase 2 gene in Belgian Parkinson's disease patients. Eur. J. Hum. Genet. **16**, 471–479.

18 Paisán-Ruíz, C., Jain, S., Evans, E. W., Gilks, W. P., Simón, J., van der Brug, M., De Munain, A. L., Aparicio, S., Gil, A. M., Khan, N., et al. (2004) Cloning of the gene containing mutations that cause PARK8-linked Parkinson's disease. Neuron **44**, 595–600.

19 Aasly, J. O., Vilariño-güell, C., Dachsel, J. C., Philip, J., West, A. B., Haugarvoll, K., Krisztina, K., Toft, M., Nutt, J. G., Payami, H., et al. (2010) Novel Pathogenic Lrrk2 p.Asn1437His substitution in familial Parkinson's disease. Mov. Disord. **25**, 2156–2163.

20 Mata, I. F., Davis, M. Y., Lopez, A. N., Dorschner, M. O., Martinez, E., Yearout, D., Cholerton, B. A., Hu, S. C., Edwards, K. L., Bird, T. D., et al. (2016) The discovery of LRRK2 p.R1441S, a novel mutation for Parkinson's disease, adds to the complexity of a mutational hotspot. Am. J. Med. Genet. Part B Neuropsychiatr. Genet., Blackwell Publishing Inc. **171**, 925–930.

21 Mata, I. F., Kachergus, J. M., Taylor, J. P., Lincoln, S., Aasly, J., Lynch, T., Hulihan, M. M., Cobb, S. A., Wu, R. M., Lu, C. S., et al. (2005) LRRK2 pathogenic substitutions in Parkinson's disease. Neurogenetics **6**, 171–177.

22 Huang, Y., Halliday, G. M., Vandebona, H., Mellick, G. D., Mastaglia, F., Stevens, J., Kwok, J., Garlepp, M., Silburn, P. A., Horne, M. K., et al. (2007) Prevalence and clinical features of common LRRK2 mutations in Australians with Parkinson's disease. Mov. Disord. **22**, 982–989.

23 Yonova-Doing, E., Atadzhanov, M., Quadri, M., Kelly, P., Shawa, N., Musonda, S. T. S., Simons, E. J., Breedveld, G. J., Oostra, B. A. and Bonifati, V. (2012) Analysis of LRRK2, SNCA, Parkin, PINK1, and DJ-1 in Zambian patients with Parkinson's disease. Park. Relat. Disord., Elsevier Ltd **18**, 567–571.

24 Janković, M. Z., Kresojević, N. D., Dobričić, V. S., Marković, V. V., Petrović, I. N., Novaković, I. V. and Kostić, V. S. (2015) Identification of novel variants in LRRK2 gene in patients with Parkinson's disease in Serbian population. J. Neurol. Sci. **353**, 59–62.

25 Toft, M., Mata, I. F., Ross, O. A., Kachergus, J., Hulihan, M. M., Haugarvoll, K., Stone, J. T., Blazquez, M., Gibson, J. M., Aasly, J. O., et al. (2007) Pathogenicity of the LRRK2 R1514Q substitution in Parkinson's disease. Mov. Disord. **22**, 389–392.

26 Pchelina, S. N., Yakimovskii, A. F., Emelyanov, A. K., Ivanova, O. N., Schwarzman, A. L. and Singleton, A. B. (2008) Screening for LRRK2 mutations in patients with Parkinson's disease in Russia: Identification of a novel LRRK2 variant. Eur. J. Neurol. **15**, 692–696.

27 Bryant, N., Malpeli, N., Ziaee, J., Blauwendraat, C., Liu, Z. and West, A. B. (2021) Identification of LRRK2 missense variants in the accelerating medicines partnership Parkinson's disease cohort. Hum. Mol. Genet. **30**, 454–466.

28 Paisán-Ruíz, C., Evans, E. W., Jain, S., Xiromerisiou, G., Gibbs, J. R., Eerola, J., Gourbali, V., Hellström, O., Duckworth, J., Papadimitriou, A., et al. (2006) Testing association between LRRK2 and Parkinson's disease and investigating linkage disequilibrium. J. Med. Genet. **43**, 1–5.

29 Shojaee, S., Fazlali, Z., Ghazavi, F., Banihosseini, S. S., Kazemi, M. H., Parsa, K., Sadeghi, H., Sina, F., Shahidi, G. A., Ronaghi, M., et al. (2009) Identification of four novel potentially Parkinson's disease associated LRRK2 variations among Iranian patients. Neurosci. Lett. **467**, 53–57.

Supplementary Table 1

30 Lorenzo-Betancour, O., Samaranch, L., Ezquerra, M., Tolosa, E., Lorenzo, E., Irigoyen, J., Gaig, C., Pastor, M. A., Soto-Ortolaza, A. I., Ross, O. A., et al. (2012) LRRK2 Haplotype-Sharing Analysis in Parkinson's Disease Reveals a Novel p.S1761R Mutation. Mov. Disord. **27**, 146–151.

31 Khan, N. L., Jain, S., Lynch, J. M., Pavese, N., Abou-Sleiman, P., Holton, J. L., Healy, D. G., Gilks, W. P., Sweeney, M. G., Ganguly, M., et al. (2005) Mutations in the gene LRRK2 encoding dardarin (PARK8) cause familial Parkinson's disease: Clinical, pathological, olfactory and functional imaging and genetic data. Brain **128**, 2786–2796.

32 Lesage, S., Janin, S., Lohmann, E., Leutenegger, A. L., Leclere, L., Viallet, F., Pollak, P., Durif, F., Thobois, S., Layet, V., et al. (2007) LRRK2 exon 41 mutations in sporadic Parkinson disease in Europeans. Arch. Neurol. **64**, 425–430.

33 Lu, C. S., Simons, E. J., Wu-Chou, Y. H., Fonzo, A. Di, Chang, H. C., Chen, R. S., Weng, Y. H., Rohé, C. F., Breedveld, G. J., Hattori, N., et al. (2005) The LRRK2 I2012T, G2019S, and I2020T mutations are rare in Taiwanese patients with sporadic Parkinson's disease. Park. Relat. Disord. **11**, 521–522.

34 Di Fonzo, A., Rohé, C. F., Ferreira, J., Chien, H. F., Vacca, L., Stocchi, F., Guedes, L., Fabrizio, E., Manfredi, M., Vanacore, N., et al. (2005) A frequent LRRK2 gene mutation associated with autosomal dominant Parkinson's disease. Lancet **365**, 412–415.

35 Gilks, W., Abousleiman, P., Gandhi, S., Jain, S., Singleton, A., Lees, A., Shaw, K., Bhatia, K., Bonifati, V. and Quinn, N. (2005) A common mutation in idiopathic Parkinson's disease. Lancet **365**, 415–416.

36 Nichols, W., Pankratz, N., Hernandez, D., Paisan-Ruiz, C., Jain, S., Halter, C., Michaels, V., Reed, T., Rudolph, A. and Shults, C. (2005) Genetic screening for a single common mutation in familial Parkinson's disease. Lancet **365**, 410–412.

37 Funayama, M., Hasegawa, K., Ohta, E., Kawashima, N., Komiyama, M., Kowa, H., Tsuji, S. and Obata, F. (2005) An LRRK2 mutation as a cause for the Parkinsonism in the original PARK8 family. Ann. Neurol. **57**, 918–921.

38 Nichols, R. J., Dzamko, N., Morrice, N. A., Campbell, D. G., Deak, M., Ordureau, A., Macartney, T., Tong, Y., Shen, J., Prescott, A. R., et al. (2010) 14-3-3 Binding to LRRK2 is disrupted by multiple Parkinson's disease-associated mutations and regulates cytoplasmic localization. Biochem. J. **430**, 393–404.

39 Di Fonzo, A., Wu-Chou, Y. H., Lu, C. S., Van Doeselaar, M., Simons, E. J., Rohé, C. F., Chang, H. C., Chen, R. S., Weng, Y. H., Vanacore, N., et al. (2006) A common missense variant in the LRRK2 gene, Gly2385Arg, associated with Parkinson's disease risk in Taiwan. Neurogenetics **7**, 133–138.

40 Clarimón, J., Pagonabarraga, J., Paisán-Ruíz, C., Campolongo, A., Pascual-Sedano, B., Martí-Massó, J. F., Singleton, A. B. and Kulisevsky, J. (2008) Tremor dominant Parkinsonism: Clinical description and LRRK2 mutation screening. Mov. Disord. **23**, 518–523.

41 Kessler, C., Atasu, B., Hanagasi, H., Simón-Sánchez, J., Hauser, A. K., Pak, M., Bilgic, B., Erginel-Unaltuna, N., Gurvit, H., Gasser, T., et al. (2018) Role of LRRK2 and SNCA in autosomal dominant Parkinson's disease in Turkey. Park. Relat. Disord. **48**, 34–39.

42 Dzamko, N., Deak, M., Hentati, F., Reith, A. D., Prescott, A. R., Alessi, D. R. and Nichols, R. J. (2010) Inhibition of LRRK2 kinase activity leads to dephosphorylation of Ser 910/Ser935, disruption of 14-3-3 binding and altered cytoplasmic localization. Biochem. J. 430, 405–413.
